# TGF-beta and TNF-alpha cooperatively induce mesenchymal transition of lymphatic endothelial cells via activation of Activin signals

**DOI:** 10.1371/journal.pone.0232356

**Published:** 2020-05-01

**Authors:** Yasuhiro Yoshimatsu, Shiori Kimuro, Joris Pauty, Kazuki Takagaki, Sanae Nomiyama, Akihiko Inagawa, Kentaro Maeda, Katarzyna A. Podyma-Inoue, Kentaro Kajiya, Yukiko T. Matsunaga, Tetsuro Watabe

**Affiliations:** 1 Department of Biochemistry, Graduate School of Medical and Dental Sciences, Tokyo Medical and Dental University (TMDU), Tokyo, Japan; 2 Laboratory of Oncology, School of Life Sciences, Tokyo University of Pharmacy and Life Sciences, Tokyo, Japan; 3 Division of Pharmacology, Graduate School of Medical and Dental Sciences, Niigata University, Niigata, Japan; 4 Institute of Industrial Science, The University of Tokyo, Tokyo, Japan; 5 Shiseido Global Innovation Center, Yokohama, Japan; Osaka University, JAPAN

## Abstract

Lymphatic systems play important roles in the maintenance of fluid homeostasis and undergo anatomical and physiological changes during inflammation and aging. While lymphatic endothelial cells (LECs) undergo mesenchymal transition in response to transforming growth factor-β (TGF-β), the molecular mechanisms underlying endothelial-to-mesenchymal transition (EndMT) of LECs remain largely unknown. In this study, we examined the effect of TGF-β2 and tumor necrosis factor-α (TNF-α), an inflammatory cytokine, on EndMT using human skin-derived lymphatic endothelial cells (HDLECs). TGF-β2-treated HDLECs showed increased expression of SM22α, a mesenchymal cell marker accompanied by increased cell motility and vascular permeability, suggesting HDLECs to undergo EndMT. Our data also revealed that TNF-α could enhance TGF-β2-induced EndMT of HDLECs. Furthermore, both cytokines induced the production of Activin A while decreasing the expression of its inhibitory molecule Follistatin, and thus enhancing EndMT. Finally, we demonstrated that human dermal lymphatic vessels underwent EndMT during aging, characterized by double immunostaining for LYVE1 and SM22α. These results suggest that both TGF-β and TNF-α signals play a central role in EndMT of LECs and could be potential targets for senile edema.

## Introduction

Blood vessels supply oxygen and nutrition to the whole body through circulating system. In peripheral tissues, the capillaries exchange their content by moving fluid from the capillaries into an interstitial compartment. Lymphatic vessels are responsible for moving water (fluid), proteins as well as lipids and immune cells from the interstitium of parenchymal organs to the draining lymph nodes and veins. Thus together with blood vessels, lymphatic vessels play an important role in maintaining homeostasis of body fluids and immune functions. The importance of the roles performed by the lymphatics is highlighted by the extensive tissue edema and impaired immunity observed in mice and humans with decreased lymphangiogenesis [1] or surgical removal of draining lymph nodes [2]. The lymphatic functions are regulated by physiological and pathological conditions including inflammation and cancer. Furthermore, recent reports have demonstrated that aging leads to anatomical and functional changes of lymphatics [3, 4], which are associated with decreased lymphatic contractility and flow [4–6].

Lymphatic vessels can be classified into two groups; initial lymphatics (lymphatic capillaries) responsible for collecting tissue fluids in peripheral tissues and collecting lymphatics which carry lymph to veins. Endothelium of blood vessels forms a highly restrictive barrier to prevent leakage of blood. The endothelial barrier dysfunction is related to various pathological conditions including inflammation. Endothelium of initial lymphatic vessels has a relatively loose structure, while collecting lymphatic vessels form a very tight endothelial barrier. Blood and lymphatic vessels are composed of a single layer of endothelial cells which line their lumens, blood vascular endothelial cells (BECs) and lymphatic endothelial cells (LECs), respectively. The barrier function of the endothelial cell monolayer is governed mainly by cell-cell contacts of endothelial cells. These characteristics of endothelial cells are prone to change in response to various extracellular cues including inflammatory cytokines.

Endothelial-to-mesenchymal transition (EndMT) is a phenomenon in which endothelial cells lose their cellular features while acquiring properties of mesenchymal cells, including loss of cell-cell contacts, decreased expression of endothelial cell marker genes, increased expression of mesenchymal cell markers and high motility. EndMT has been reported to take place during embryonic cardiac valve formation [7]. However, many reports also point an important role of EndMT in progression of various diseases, including cancer [8], pulmonary arterial hypertension [9] and fibrosis [10]. EndMT can be induced by various growth factors and inflammatory cytokines, among which transforming growth factor-β (TGF-β) plays a major role [11].

TGF-β is a multifunctional cytokine that has been shown to regulate various biological events such as cell proliferation, differentiation and apoptosis [12]. The TGF-β initiates signal by binding to its two serine-threonine kinase type receptors, TGF-β type I receptor (TβR-I)/ALK5 and TGF-β type II receptor (TβR-II). Activated ALK5 phosphorylates Smad2/3, an intracellular signal transduction factor, that results in a formation of a transcriptional complex with co-Smad factor, Smad4, their subsequent translocation into the nucleus and stimulation of the expression of a number of direct target genes, e.g., TMEPAI, Smad7 and PAI-1. Smad2/3 signals are also activated by other members of TGF-β family including Activin and Nodal, that transduce their signals via ALK4 and ALK7 type I receptors, respectively [12]. However, TGF-β signals are not mediated only by Smads. There are also other, Smad-independent, so called non-Smad pathways, that involve the Mitogen-activated Protein Kinase (MAPK) signaling pathway or Rho/ROCK signaling pathway and that have been shown to participate in transducing TGF-β signals. Interestingly, both types of signaling pathways have been reported to control EndMT [11].

We have previously reported that TGF-β signal suppresses proliferation of human dermal LECs (HDLECs) and inhibits lymphangiogenesis [13]. The growth inhibitory effects of TGF-β signals on LECs are caused by the decreased expression of Prox1 transcription factor by TGF-β [13]. Prox1 is important for the proliferation of HDLECs since decreased Prox1 expression by siRNA suppressed their proliferation [14]. Furthermore, Prox1 is necessary for the maintenance of LEC identity since deletion of Prox1 also results in a loss of LEC characteristics and a dysfunction of lymphatic vessels [15, 16]. In addition, it has also been shown that the properties of LECs can also be affected by TGF-β treatment, resulting in EndMT [17]. Interestingly, this effect of TGF-β can be counteracted by Fibroblast growth factor (FGF) signal, which seems to play a pivotal role in maintaining LEC identity [17].

Effects of TGF-β on the mesenchymal transition of epithelial cells are regulated by various cytokines. We have previously reported that TGF-β-induced epithelial-to-mesenchymal transition (EMT) of A549 lung cancer cells can be augmented by tumor necrosis factor-α (TNF-α) [18], resulting in loss of their adhesive properties and gain of migratory and invasive abilities characteristic to mesenchymal cells. TNF-α is a pro-inflammatory cytokine which increases vascular leakage and permeability of endothelial monolayer [19, 20]. Pro-inflammatory cytokines including TNF-α and interleukin-1β (IL-1β) have been reported to induce EndMT of various types of endothelial cells [21–23].

Inflammation shares multiple characteristics with aging and age-related diseases. During aging, chronic, sterile and low-grade inflammation, termed inflammaging, develops [24]. Since inflammaging is a risk factor for age-related diseases, identification of pathways that control age-related inflammation across multiple systems including vascular systems is critical in order to elucidate whether therapies against inflammaging may be beneficial in elderly people.

Although several studies have reported the molecular mechanism underlying EndMT of LECs, the details regarding EndMT of LECs largely remain unclear. Thus in the present study, we attempted to elucidate the roles of TGF-β and TNF-α in the regulation of mesenchymal transition of LECs.

## Materials and methods

### Cells culture and reagents

HDLECs were purchased from Lonza (Basel, Switzerland). The cells were maintained in EBM ^TM^-2MV Bullet Kit (cc-3202, Lonza) and used for study after extensive characterization. HEK-Blue^TM^ TGF-β cells (HEK293 cell-derived TGF-β responsive reporter cells) were purchased from InvivoGen (San Diego, CA, USA) and maintained in Dulbecco’s Modified Eagle’s Medium (Nacalai Tesque: Kyoto, Japan) supplemented with 10% Fetal Bovine Serum (FBS, SIGMA: St. Louis, MO, USA), 100 units/mL Penicillin-100 μg/mL Streptomycin (Nacalai Tesque). TGF-β2, TNF-α, and Activin A were purchased from Peprotech (Rocky Hill, NJ, USA). Follistatin was purchased from R&D Systems (Minneapolis, MN, USA). SB431542 and Y27632 were obtained from WAKO Chemicals (Saitama, Japan). Concentrations of TGF-β2 used were determined empirically, based on their ability to alter expression of various endothelial and mesenchymal markers.

### RNA interference

The siRNAs for human SMAD2 (#A: 5’- CAAGUACUCCUUGCUGGAUTT -3’, #B: 5’- GUCCCAUGAAAAGACUUAATT -3’) and human MRTF-A (#A: 5’- GUGUCUUGGUGUAGUGUAATT -3’, #B: 5’-CAGGUGAACUAUCCCAAAGUATT -3’) were synthesized by Hokkaido System Science (Sapporo, Japan). The target sequences for SMAD2 were designed as previously described [25]. Negative control was purchased from QIAGEN (Hilden, Germany). All siRNAs were introduced into the cells using Lipofectamine RNAi Max reagent (Invitrogen: Carlsbad, CA, USA) according to the manufacturer's instructions. Briefly, 8.0×10^4^ cells were mixed with siRNA-Lipofectamine complexes and seeded into 6-well plates. Medium was refreshed 6 h later and cells were allowed to grow for an indicated period.

### RNA isolation and quantitative RT-PCR

Total RNA was prepared with Nucleospin RNA (TaKaRa Bio: Otsu, Japan) according to the protocol suggested by the manufacturer and reverse-transcribed by random priming using a PrimeScript II Kit (TaKaRa Bio). The quantitative RT-PCR (qRT-PCR) analysis was performed using StepOne Plus Real-Time PCR System (Thermo Fisher Scientific, Waltham, MA, USA). All expression data were normalized to that of β-actin. The primer sequences are available in Table 1.

**Table 1:** Primers for quantitative RT-PCR.

Transcript	specific	Primer	Sequence (5’ to 3’)
*ACTB* (*β*-actin)	human/mouse	5’	TCACCCACACTGTGCCCATCTACGA
		3’	CAGCGGAACCGCTCATTGCCAATGG
*ANGPT2* (Angiopoietin2)	human	5’	ACCTGTTGAACCAAACAGCG
		3’	TTGTCGAGAGGGAGTGTTCC
*FN1* (Fibronectin1)	human	5’	AAACCAATTCTTGGAGCAGG
		3’	CCATAAAGGGCAACCAAGAG
*FST* (Follistatin)	human	5’	ACGTGTGAGAACGTGGACTG
		3’	CACATTCATTGCGGTAGGTTTTC
*ICAM1*	human	5’	TGCCATGCAGCTACACCTAC
		3’	CAGGTACCATGGCCCCAAAT
*ID1*	human	5’	AGCACGTCATCGACTACATCAGG
		3’	GGATTCCGAGTTCAGCTCCAA
*INHBA* (Inhibin βA)	human	5’	CCTCCCAAAGGATGTACCCAA
		3’	CTCTATCTCCACATACCCGTTCT
*LYVE1*	human	5’	ACCCTGGTGTTGCTTCTCACT
		3’	GGTTCGCCTTTTTGCTCACA
*MRTFA* (MRTF-A)	human	5’	CCATGGACTCATCCTACGCC
		3’	AGTGAGGTGCTGTTCTGACG
*PMEPA1* (TMEPAI)	human	5’	TTTGTGGGAATGGCTTTGCG
		3’	GAGGACACTGGGCTTCAACA
*PROX1* (Prox1)	human	5’	CCCAGGACAGTTTATTGACCGA
		3’	GGTTGTAAGGAGTTTGGCCCAT
*SMAD2* (Smad2)	human	5’	GAGTGCCTAAGTGATAGTGC
		3’	ACAGACTGAGCCAGAAGAGC
*SMAD4* (Smad4*)*	human	5’	CTTTGAGGGACAGCCATCGT
		3’	GATGGGGCTAACAGAGCTGG
*SMAD7* (Smad7)	human	5’	TGCAACCCCCATCACCTTAG
		3’	GACAGTCTGCAGTTGGTTTGAGA
*SNAI1* (Snail)	human	5’	TTCTCACTGCCATGGAATTCC
		3’	GCAGAGGACACAGAACCAGAAA
*TAGLN* (SM22α)	human	5’	TCAAGCAGATGGAGCAGGTG
		3’	GCTGCCATGTCTTTGCCTTC
*TGFB1* (TGF-β1)	human	5’	GGAAATTGAGGGCTTTCGCC
		3’	CCGGTAGTGAACCCGTTGAT
*TGFB2* (TGF-β2)	human	5’	GTTCGATTTGACGTCTCAGCAAT
		3’	CAATCCGTTGTTCAGGCACTCT
*Tgfb2* (TGF-β2)	mouse	5’	ATAATTGCTGCCTTCGCCCT
		3’	CCCCAGCACAGAAGTTAGCATT
*TGFB3* (TGF-β3)	human	5’	ATGACCCACGTCCCCTATCA
		3’	TCCGACTCGGTGTTTTCCTG

### Smad2/3-responsive reporter assay

Activation of Smad2/3 signals by TGF-β family members including TGF-β1, 2, 3, and Activin A was determined using reporter assay which employs HEK-Blue^TM^ TGF-β cells allowing direct quantification of soluble alkaline phosphatase (SEAP) expressed under control of Smad3/4-inducible elements. HDLECs were treated with TGF-β2 or SB431542 for 48 h. The culture medium was then replaced with EGM-2MV medium with 0.5% FBS and HDLECs were incubated for additional 6 h. The conditioned medium was then collected and added to the pre-seeded HEK-Blue^TM^ TGF-β reporter cells, followed by incubation for 24 h. Fraction of the medium obtained from incubation with HEK-Blue cells was then mixed with QUANTI-Blue substrate (InvivoGen) and incubated for 30 min. The released SEAP was quantified at 640 nm using spectrophotometer.

### Chamber migration assay

The modified Boyden chamber migration assay was used for the measurements of migration of HDLECs. Briefly, the chambers were pre-coated with Cellmatrix Type I-C (Nitta Gelatin: Yao, Japan). HDLECs were cultured in the absence or presence of TGF-β2 for 72 h, harvested and suspended in EGM-2MV medium. The cells were then seeded (1.0×10^4^ cells/chamber) in the upper chamber of a 24-well Transwell filter (8 μm pore filter, Merck Millipore) and allowed to migrate for 6 h. After indicated period, the filters were stained with DiffQuik (Sysmex: Kobe, Japan) followed by removal of non-migrated cells with a cotton swab. Migrated cells adhering to the bottom side of the filter were examined, photographed using a light microscope (BZ-X710, Keyence: Osaka, Japan) and counted.

### Tube formation assay

A 12-well plate was pre-coated with Matrigel (BD Biosciences, San Jose, USA) (0.4 mL/well). After polymerization of Matrigel at 37°C for 1 h, HDLECs were seeded in each well at a density of 1.75×10^5^ cells/400 μL/well. After 7 h of incubation in EGM-2MV medium supplemented with 5% FBS, tube-like structures were photographed under phase-contrast microscopy (BZ-X710, KEYENCE) with 10× objective lenses. Tube length was quantified using ImageJ (US National Institutes of Health, Bethesda, MD, USA).

### Immunocytochemistry

HDLECs were seeded on the cover glass pre-coated with Cellmatrix Type I-C (Nitta Gelatin) and cultured in the absence or presence of TGF-β2 and/or TNF-α for 72 h. The cells were then fixed with 4% paraformaldehyde, treated with 0.1% Triton X-100, blocked and incubated with primary anti-SM22α (ab14106, Abcam) and anti-Prox1 (AF2727, R&D systems) antibodies, followed by treatment with Alexa 488- and Alexa 594-conjugated secondary antibodies, respectively and Hoechst33342 for staining of the nuclei. Images were taken with All-in-One fluorescent microscope, BZ-X710 (Keyence).

### Microvasculature preparation and permeability assay

The description and fabrication method of polydimethylsiloxane (PDMS)-based chips were previously reported [26]. Briefly, the PDMS chip (25 mm × 25 mm × 5 mm: width × length × height) comprised a central rectangular chamber framed on its left and right sides by two wells and crossed by a *φ* 300-μm PDMS channel. For building the micro-lymphatic vessels (micro-LVs), the PDMS chips were first treated with O_2_ plasma. For each chip, a *φ* 200-μm needle (acupuncture needle, No.02, 0.20 mm x 30 mm, J type, from Seirin: Shizuoka, Japan) was coated with PBS, 1% BSA. The chips and needles were then sterilized by UV-light exposure under a cell culture hood. A neutralized collagen solution was prepared by mixing on ice in a 8:1:1 volume ratio: Cellmatrix Type I-A collagen solution (3 mg/mL, pH 3, from Nitta Gelatin), 10× Hank’s buffer, and 10× collagen buffer (262 mM NaHCO_3_, 20 mM HEPES, 0.05N NaOH). Ice-cold collagen solution was introduced into the wells and rectangular chamber and the BSA-coated needle was inserted through the PDMS channel. After removal of the excessive collagen from the wells, the collagen was allowed to solidify by incubating the device in a cell culture incubator for one hour (37°C, 5% CO_2_). Withdrawal of the needle left a hollow channel within the collagen gel that served as a scaffold for building a tubular lymphatic endothelium. HDLECs, treated for 24 h with normal EBM ^TM^-2MV or with EBM ^TM^-2MV containing 5 μM SB431542 or 10 ng/mL TGF-β2, were harvested and resuspended in same media at a density of 1×10^7^ cells/mL. 20,000 cells were loaded at each opening of the collagen tubular scaffold and allowed to attach for 15 minutes. Finally, 1 mL of drug-containing media was added and the micro-LVs were cultured in a cell culture incubator (37°C, 5% CO_2_) with media renewal every other day. Six days after micro-LVs fabrication, micro-LVs were imaged using a microscope Observer Z1 (Carl Zeiss: GmbH, Oberkochen, Germany) and permeability assay was performed as previously described [26] with minor changes. Briefly, the chips were set-up on a confocal microscope LSM700 (CLSM, Carl Zeiss, Jena, Germany). Medium was removed and 15 μL of a 100 μg/mL FITC-dextran (70 kDa) in EBM ^TM^-2MV solution were introduced in both wells. The fluorescence of FITC was imaged with a 488-nm-wavelength laser and an optical section of 77.8 μm. For quantifying the microvessel permeability, CLSM images taken one minute after introduction of FITC-dextran were processed with the ZEN 2012 SP1 black edition software (version 8.1.0.484, 64 bit, Carl Zeiss). The mean fluorescence intensity of FITC detected in collagen gel was given by the Zen software for two regions of interest (50 μm × 2500 μm) drew close and aligned to each edge of a microvessel. The obtained values were averaged and considered as representative of a micro-LV’s permeability to 70 kDa molecules. The experiment was performed three times with five micro-LVs. After permeability assay, total RNA was extracted from three micro-LVs and processed for qRT-PCR analyses.

### Immunohistochemistry

Human skin samples were obtained from the eyelids of 16 healthy volunteers aged 20–80 years old and were snap-frozen. Skin samples were divided into 2 groups according to age of the donor: Group 1: 12–40 (N = 8) and Group 2: over 40 (N = 10). Double immunofluorescence staining for LYVE1 and SM22α was performed as previously described [27, 28]. Frozen skin sections were fixed with 4% PFA at 4ºC for 10 min and blocked with a blocking solution for 30 min. The sections were then incubated overnight with goat anti-LYVE1 IgG (AF2089, R&D systems) and rabbit anti-SM22α IgG (ab14106, abcam: Cambridge, UK). The next day the samples were incubated with Alexa Fluor 594-conjugated donkey anti-rabbit IgG and Alexa Fluor 488-conjugated donkey anti-goat IgG (Invitrogen) diluted to 1:200 for 1 h. The pictures were captured under LSM 510 META microscope (Carl Zeiss). Images were analyzed using IP-Lab software (Scanalytics: Fairfax, VA, USA). Briefly, the relative area occupied by SM22α^+^ LYVE1^+^ vessels to the one occupied by LYVE1^+^ vessels (total lymphatic vessel area) was defined as EndMT ratio. The analyzed areas were determined in the dermis, in an area within 500 μm distance from the epidermal-dermal junction. Then the ratio of SM22α^+^/ LYVE1^+^ was calculated by dividing the area of SM22α^+^LYVE1^+^ vessels by that of LYVE1^+^ vessels per area unit (mm^2^).

### Statistical analyses

Values are presented as mean ± standard deviation (S.D.) or standard error of the mean (S.E.M.). Significant differences between means were determined using two-tailed unpaired Student's *t*-test or one/two-way ANOVA followed by Bonferroni multiple comparison *post hoc* analysis using EZR software [29]. Differences between means were considered statistically significant at *P < 0.05, **P<0.01, ***P<0.001, ****P<0.0001; N.S., not significant.

### Ethical standards

All the experiments were approved by “the Safety Control Committee for Experiments Using Genetically Modified Organisms, Etc.” and done according to the guideline of “Safety Control Regulations for Experiments Using Genetically Modified Organisms, Etc., Tokyo Medical and Dental University” (approved number: G2018-047C). Human skin samples were obtained by the Gakugeidai-Nishiguchi Clinic (Tokyo, Japan). All procedures involving human subjects were approved by the Institutional Review Board of the Shiseido Global Innovation Center, and all subjects provided written informed consent. Ethics Review Committee of Faculty of Dentistry at Tokyo Medical and Dental University (TMDU) has waived the requirement for this approval.

## Results

### TGF-β signals induced mesenchymal transition of lymphatic endothelial cells

**Fig 1:**
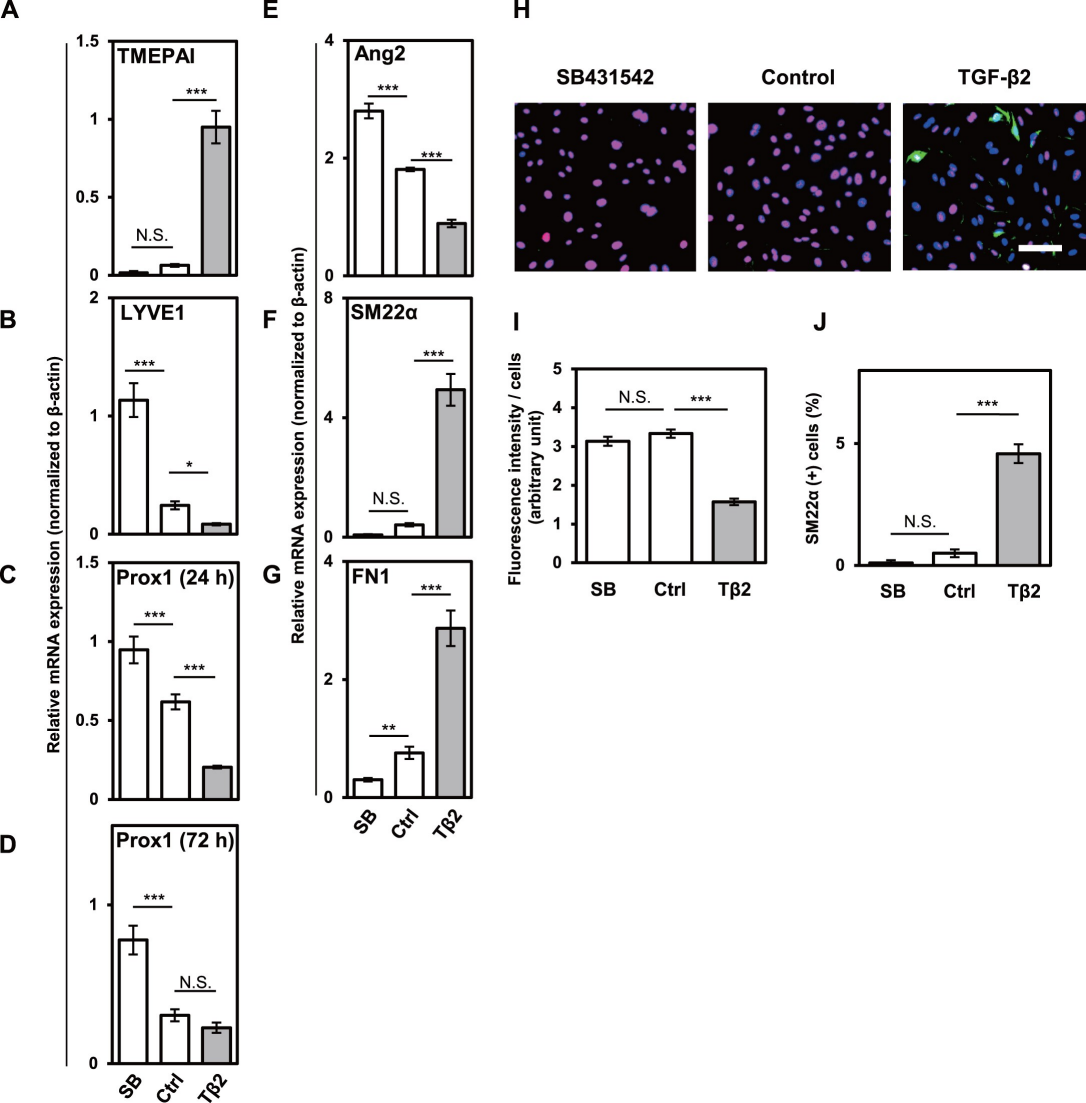
Effects of TGF-β2 and SB431542 on the lymphatic endothelial and mesenchymal characteristics of HDLECs. HDLECs were cultured in the absence (Control: Ctrl) or presence of 0.1 ng/mL of TGF-β2 (TGF-β2 or Tβ2) or 5 μM of SB431542 (SB) for 72 h (A, B, D-G) or 24 h (C), followed by qRT-PCR analyses for the expression of TMEPAI (A), LYVE1 (B), Prox1 (C, D), Ang2 (E), SM22α (F), and FN1 (G), and fluorescence immunostaining (H) for SM22α (green), Prox1 (red) and nuclei (blue). Scale bar, 200 μm. Expression of Prox1 (I) and SM22α (J) revealed by immunocytochemical analysis was quantified. Data are represented as mean ± S.D., N = 4 (A-G), and as mean± S.E.M., N = 8 (I, J), representative of three independent experiments. *P < 0.05, **P < 0.01, ***P < 0.001; N.S., not significant. Differences are tested using one-way ANOVA followed by Bonferroni multiple comparison *post hoc* analysis.

In order to examine the effects of TGF-β signal on the expression of various EndMT-related markers in LECs, we treated HDLECs with TGF-β2 or SB431542, an inhibitor of ALK4/5/7 type I receptors, for 72 h (Fig 1). TGF-β2 significantly increased while SB431542 had little effect on the expression of TMEPAI, a direct target gene of TGF-β signals [30] in HDLECs (Fig 1A), suggesting that TGF-β signals are elevated only by exogenous TGF-β family ligands. In accordance with our previous report [13], TGF-β treatment decreased the expression of lymphatic endothelial markers, LYVE1 at 72 h (Fig 1B), and Prox1 at 24 h (Fig 1C), but did not have significant effect on Prox1 expression at 72 h (Fig 1D). Prox1 transcription factor plays important roles in the differentiation and maintenance of LECs [15, 16]. The HDLECs cultured in the presence of TGF-β2 showed a decreased expression of Angiopoietin 2 (Ang2), a lymphatic endothelial marker and target of Prox1 (Fig 1E), suggesting that the loss of LEC identity in response to TGF-β signals was at least partially mediated by TGF-β-induced decrease of Prox1 expression.

To assess whether TGF-β-driven loss of LEC identity induces mesenchymal transition in HDLECs, we examined the effects of TGF-β signals on the expression of various mesenchymal markers. We observed an increased expression of mesenchymal markers, SM22α (Fig 1F) and Fibronectin 1 (FN1) (Fig 1G) in TGF-β2-treated HDLECs, when compared to the control. The effects of TGF-β2 on the mRNA expression of lymphatic endothelial and mesenchymal markers were also confirmed at protein levels by fluorescence immunocytochemistry using antibodies for Prox1 and SM22α (Fig 1H–1J).

In order to generalize our findings, we utilized another type of LECs, human lung lymphatic microvascular endothelial cells (HMVEC-LLy) and showed that TGF-β2 elicited essentially similar effects on cell number (S1A Fig) and the expression of various lymphatic endothelial (S1B–S1D Fig) and mesenchymal markers (S1E and S1F Fig). These results suggested that upon activation of TGF-β signals, multiple types of LECs lose their endothelial phenotypes while acquiring mesenchymal characteristics.

We have previously reported that Prox1 expression in HDLECs was decreased by bone morphogenetic protein-9 (BMP-9), which is a member of TGF-β family [14]. BMPs activate intracellular signaling pathways mediated by Smad1/5/8 while TGF-βs activate Smad2/3 signals. Our present findings showing that Prox1 expression decreased upon TGF-β2 treatment prompted us to investigate the effects of TGF-β in combination with BMP-9 on the expression of various EndMT markers (S2 Fig). We found that the expression of ID1, a faithful target of Smad1/5/8 signals, was elevated in the presence of BMP-9 while this BMP-9-induced expression of ID1 was not altered by TGF-β2 (S2A Fig). On the other hand, TGF-β2-induced expression of TMEPAI was enhanced by BMP-9 (S2B Fig), suggesting that BMP-9 activates Smad2/3 signals in HDLECs. TGF-β2-induced decrease in the expression of LYVE1 (S2C Fig) and Prox1 (S2D Fig) and increase in FN1 (S2E Fig) expression were further enhanced by BMP-9 while BMP-9 did not elicit the similar effect on the expression of Ang2 (S2F Fig) and SM22α (S2G Fig). These results suggest that BMP-9 enhances TGF-β-induced EndMT in HDLECs while having differential effects on some EndMT markers including Ang2 and SM22α.

### TGF-β increased motility and decreased tube forming ability of lymphatic endothelial cells

**Fig 2:**
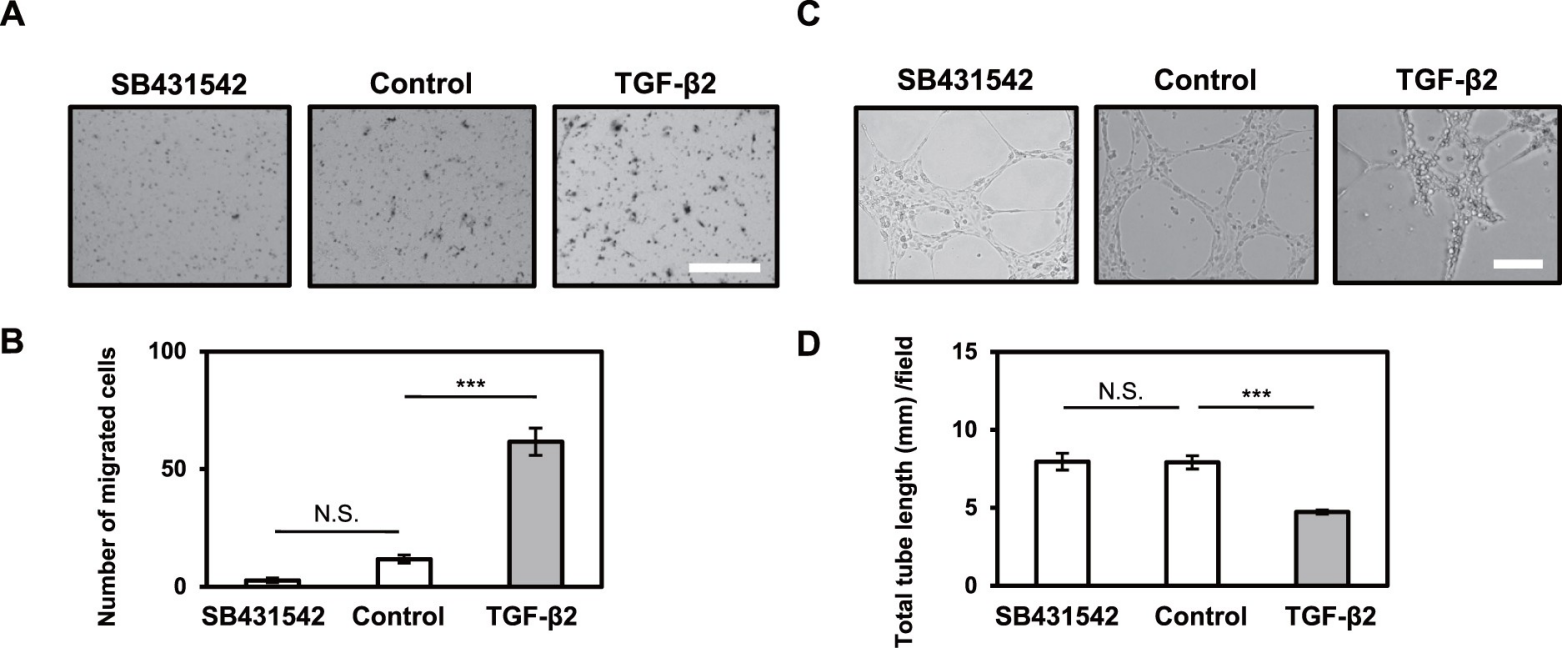
Effects of TGF-β2 and SB431542 on the migration and formation of tube-like structures of HDLECs. HDLECs were cultured in the absence (Control) or presence of 1 ng/mL (A, B) or 0.1 ng/mL (C, D) of TGF-β2 or 5 μM of SB431542 for 72 h, followed by chamber migration assay (A, B) and tube formation assay (C, D). (A, B) Cells were allowed to migrate for 6 h. The cells migrated to the bottom side of the chamber were stained (A) and counted under a phase-contrast microscope (B). Scale bar, 300 μm. (C, D) Cells were allowed to form tube-like structures on a collagen gel for 7 h, followed by phase-contrast imaging (C) and quantification (D) of tube-like structures. Scale bar, 200 μm. Data are represented as mean ± S.E.M., N = 3 (B), N = 6 (D), representative of three independent experiments. **P < 0.01, ***P < 0.001; N.S., not significant. Differences are tested using one-way ANOVA followed by Bonferroni multiple comparison *post hoc* analysis.

TGF-β induces mesenchymal transition of epithelial cells, termed EMT [31]. One of the mesenchymal properties conferred to the TGF-β-treated epithelial cells is an increased cell motility. In order to study the functional characteristics accompanied by TGF-β-induced EndMT of HDLECs, we examined the effect of TGF-β signals on HDLEC motility using chamber migration assay. As shown in Fig 2A and 2B, activation of TGF-β signals increased the motility of HDLECs, suggesting that TGF-β-treated LECs acquire mesenchymal properties.

In addition, HDLECs exhibited tube (cord) forming ability, which is characteristics of endothelial cells (Fig 2C and 2D). While TGF-β2 decreased the tube forming ability of HDLECs, SB431542 maintained their endothelial characteristics. Taking together the results of molecular biology and phenotypic analyses, we have concluded that HDLECs undergo EndMT upon TGF-β treatment with loss of endothelial and gain of mesenchymal characteristics.

### TGF-β alters the integrity of micro-lymphatic vessels

**Fig 3:**
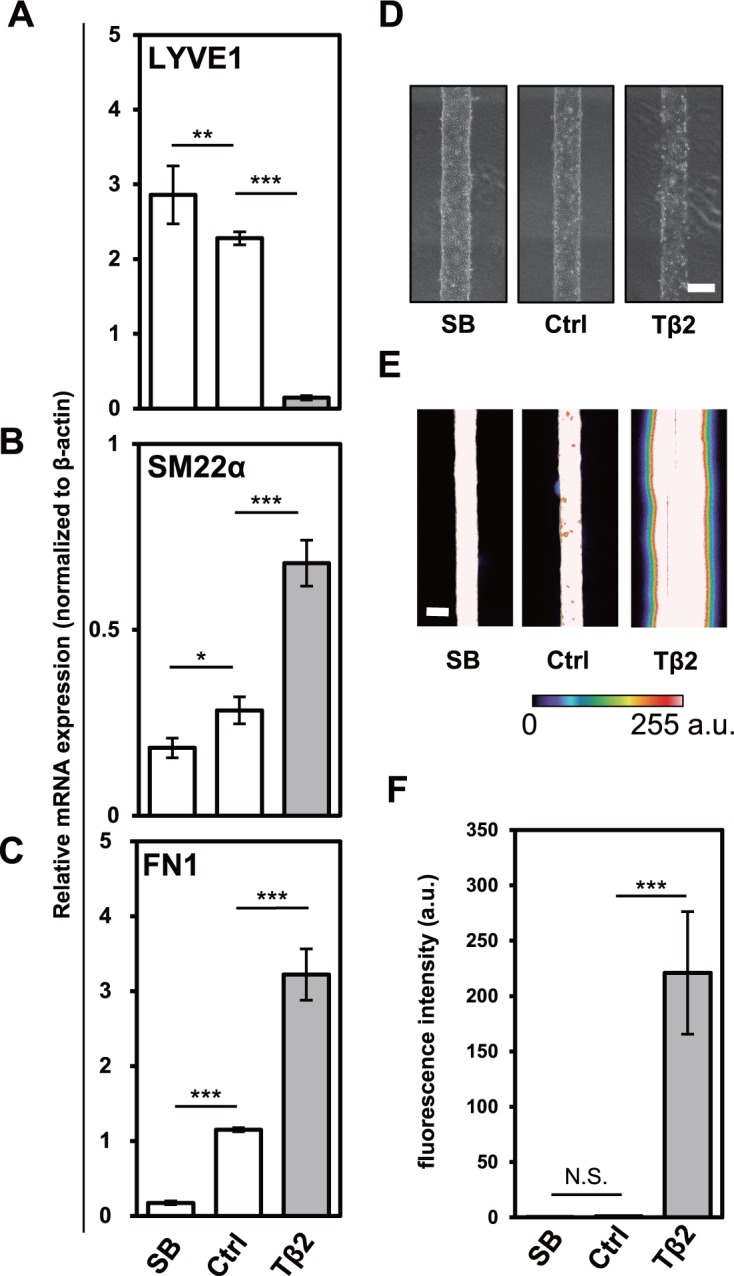
Effects of TGF-β2 and SB431542 on the formation of HDLEC-derived microvessels and their permeability. HDLECs were preincubated in the absence (Ctrl) or presence of 10 ng/mL of TGF-β2 (Tβ2) or 5 μM of SB431542 (SB) for 24 h, followed by microvessel formation in a collagen gel, and treatment with or without TGF-β2 or SB431542 for 6 days. The formed microvessels were subjected to qRT-PCR analysis for the expression of LYVE1 (A), SM22α (B) and FN1 (C), to phase-contrast imaging (D), and to permeability assay using 70 kDa FITC-dextran. Intensity of FITC fluorescence in images is shown in the colorimetric scale (0 to 255, arbitrary unit) (E, F). Leakage and diffusion of FITC-dextran into collagen gel were visualized and analyzed using ZEN software (F). Scale bar, 200 μm. Data are represented as mean ± S.D., N = 4 (A-C), N = 5 (F), representative of three independent experiments. *P < 0.05, **P < 0.01, ***P < 0.001; N.S., not significant. Differences are tested using one-way ANOVA followed by Bonferroni multiple comparison *post hoc* analysis.

Vascular barrier of the endothelium plays important roles in the maintenance of blood vessels. Dysfunction of vascular barrier has been observed under various pathological conditions and has found to be associated with many diseases. The barrier function of the endothelial cell monolayer is governed by cell-cell, cell-extracellular matrix contacts, and exogenous stimuli by various inflammatory cytokines. In order to measure the vascular permeability induced by those cytokines, we have developed a three-dimensional (3D) vascular endothelium *in vitro* model in which we could manipulate the endothelial barrier function and its permeability [26, 32]. Recent lines of evidence have shown that EndMT impairs the barrier function of blood vascular endothelial cells [33]. In order to examine the effects of TGF-β on the permeability of lymphatic vessels, we adapted our *in vitro* model technology to create a three-dimensional lymphatic endothelium by using HDLECs that were pre-treated with TGF-β2 or SB431542 for 24 h (Fig 3).

First, we examined whether EndMT could be induced in HDLECs in this *in vitro* model. HDLECs cultured in the presence of TGF-β2 for 7 days, including 6 days as micro-lymphatic vessels (micro-LVs), showed decreased expression of the LEC marker LYVE1 (Fig 3A) and increased expression of mesenchymal cell markers, SM22α (Fig 3B) and FN1 (Fig 3C), when compared to the control. At the same time, inhibition of TGF-β signals with SB431542 had an opposite effect (Fig 3A–3C).

At a tissue (3D) level, TGF-β impaired the formation of micro-LVs (Fig 3D). Interestingly, 24 h after seeding of HDLEC into the collagen gel scaffold, TGF-β2-treated HDLECs successfully formed a tubular endothelium; however, this endothelium degenerated in the successive days. This observation suggested that the effect of TGF-β2 was progressive or further sustained through time and that, as HDLECs underwent EndMT, they could no longer participate in endothelium maintenance. When assessing the endothelial barrier function by using fluorescein isothiocyanate (FITC)-conjugated dextran (70 kDa, FITC-dextran) and confocal fluorescence microscopy, we found that the barrier function of micro-LVs was slightly increased when TGF-β signals was inhibited with SB431542 (Fig 3E and 3F). On the other hand, as TGF-β2-treated HDLECs did not maintain the endothelial barrier (Fig 3D), FITC-dextran freely diffused into the collagen gel (Fig 3E and 3F). Taken together, these results suggest that TGF-β impairs barrier function of lymphatic vessels by inducing EndMT of HDLECs.

### Smad2 is indispensable for TGF-β-induced EndMT of HDLECs

**Fig 4:**
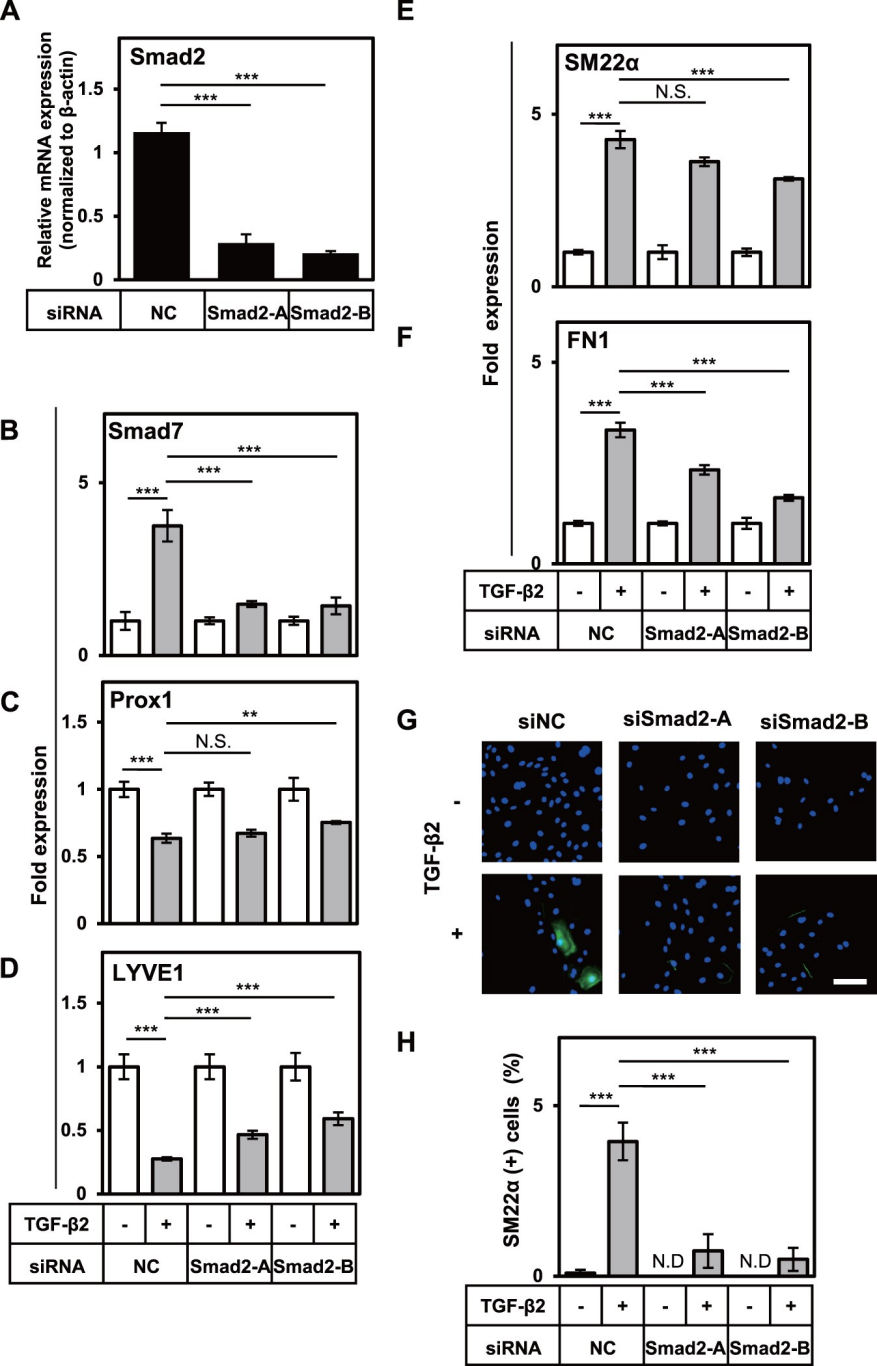
Roles of Smad2 in the TGF-β-induced changes in lymphatic endothelial and mesenchymal characteristics of HDLECs. HDLECs transfected with negative control siRNA (NC) or siRNAs for Smad2 (Smad2-A and Smad2-B) were cultured in the absence (-) or presence (+) of 0.1 ng/mL of TGF-β2 for 48 or 72 h, followed by qRT-PCR analyses for expression of Smad2 (A), Smad7 (B), Prox1 (C), LYVE1 (D), SM22α (E) and FN1 (F) and fluorescence immunostaining (G) for SM22α (green) and nuclei (blue). Scale bar, 100 μm. The numbers of SM22α-positive cells were quantified (H). Data are represented as mean± S.D., N = 4 (A-F), and as mean± S.E.M., N = 3 (H), representative of three independent experiments. **P < 0.01, ***P < 0.001; N.D., not detected, N.S., not significant. Differences are tested using one-way ANOVA (A) or two-way ANOVA (B-F, H) followed by Bonferroni multiple comparison *post hoc* analysis.

Our data suggested that TGF-β induced EndMT of HDLECs, thus in the next step we investigated signaling pathways regulating this event. TGF-β transduces intracellular signals through the formation of ALK5/TβR-II receptor complex and activation of downstream effector molecules. TGF-β signal pathways are classified into Smad-mediated pathways (Smad pathways) and Smad-independent pathways (non-Smad pathways). In order to examine whether Smad pathways are involved in the EndMT of HDLECs, we used siRNAs specific for Smad2, an essential signaling factor in the TGF-β/Smad pathway. Introduction of Smad2 siRNAs into HDLECs resulted in more than 70% decrease of Smad2 expression (Fig 4A) and decreased the expression of its target gene, Smad7 (Fig 4B). The TGF-β-induced decrease in Prox1 expression was not altered by Smad2 knockdown (Fig 4C) but the decrease in LYVE1 expression was partly suppressed by Smad2 knockdown (Fig 4D). While introduction of Smad2 siRNAs only slightly suppressed the TGF-β-induced increase in the expression of SM22α (Fig 4E) we could observe significant decrease in the expression of FN1 (Fig 4F). Furthermore, the suppression of TGF-β-dependent increase in the expression of SM22α by Smad2 knockdown was well correlated when the number of SM22α-positive cells detected by immunocytochemistry using anti-SM22α antibody (Fig 4G and 4H). These results suggest that Smad2 is indispensable for TGF-β-induced alteration in the expression of LYVE1, SM22α, and FN1 in HDLECs.

Smad2 forms multimeric complexes with Smad4 to mediate TGF-β signals. In order to further study the significance of Smad pathway in the induction of EndMT, we examined the role of Smad4 by knocking it down with specific siRNA. Introduction of two different Smad4 siRNAs into HDLECs resulted in approximately 80% and 60% decrease of Smad4 expression, respectively (S3A Fig). Smad4 knockdown was also associated with decreased expression of its target gene, TMEPAI (S3B Fig). The TGF-β-induced decrease in LYVE1 expression observed in control cells was partially suppressed by Smad4 knockdown (S3 and 4C Figs). On the other hand, TGF-β-induced, 4D Fig) was significantly reduced by Smad4 knockdown. Taken together with the results in Fig 4, our data suggest that Smad pathway is indispensable for TGF-β-induced EndMT in HDLECs.

### Rho/ROCK/MRTF-A axis is indispensable for TGF-β-induced EndMT of HDLECs

**Fig 5:**
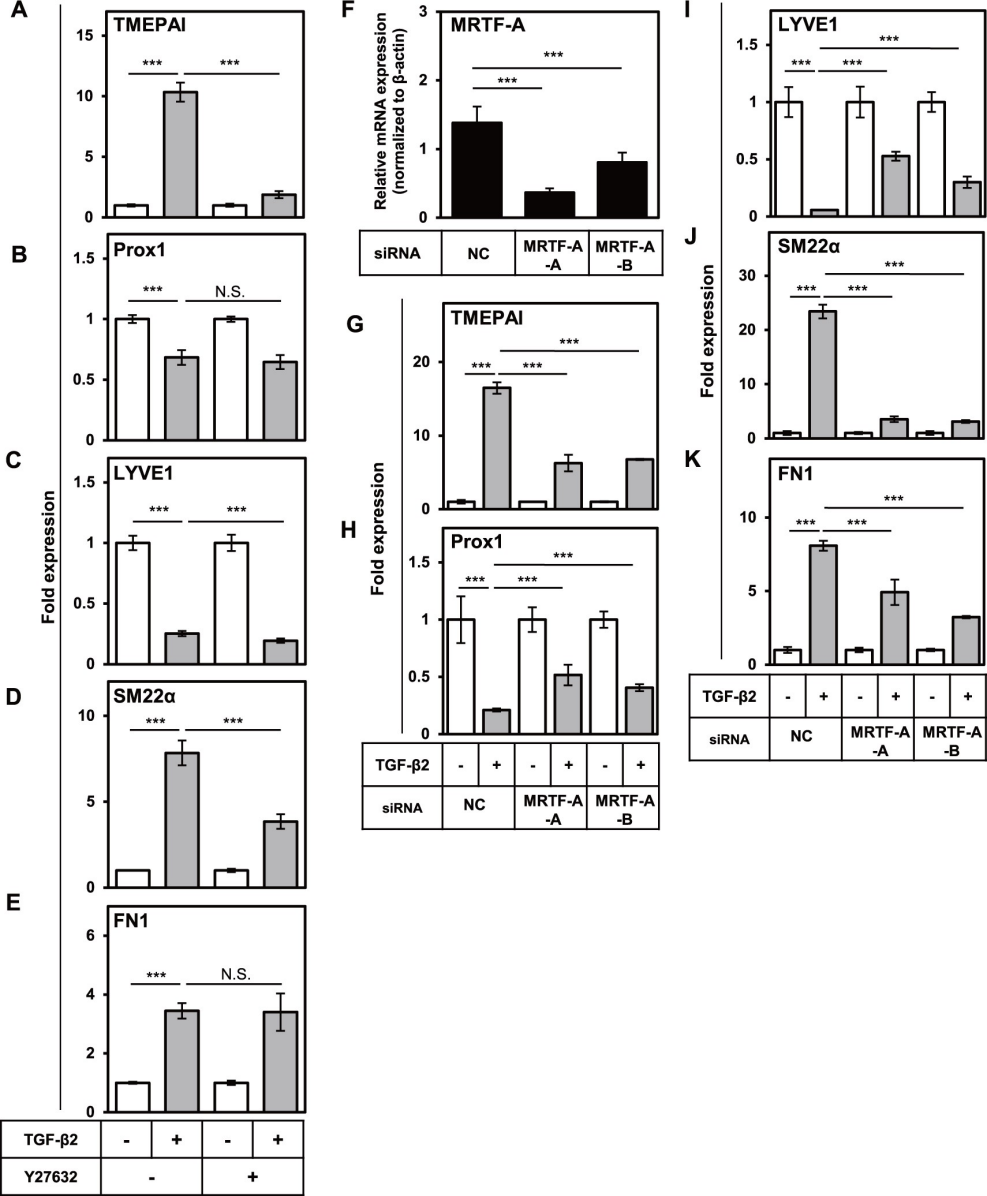
Roles of Rho/ROCK pathway and MRTF-A in the TGF-β-induced changes in lymphatic endothelial and mesenchymal characteristics of HDLECs. (A-E) HDLECs were cultured in the absence (-) or presence (+) of 0.1 ng/ml of TGF-β2 in combination with 10 μM of Y27632 for 72 h, followed by qRT-PCR analyses for expression of TMEPAI (A), Prox1 (B), LYVE1 (C), SM22α (D), and FN1 (E). (F-K) HDLECs transfected with negative control siRNA (NC) or siRNAs for MRTF-A (MRTF-A-A and MRTF-A-B) were cultured in the absence (-) or presence (+) of 1.0 ng/ml of TGF-β2 for 72 h, followed by qRT-PCR analyses for expression of MRTF-A (F), TMEPAI (G), Prox1 (H), LYVE1 (I), SM22α (J), and FN1 (K). Data are represented as mean ± S.D., N = 4, representative of three independent experiments. ***P < 0.001; N.S., not significant. Differences are tested using two-way ANOVA (A-E, G-K) or one-way ANOVA (F) followed by Bonferroni multiple comparison *post hoc* analysis.

TGF-β activates not only Smad pathway but also non-Smad pathways including Rho/ROCK pathway. We previously reported that TGF-β induced EndMT in a blood vascular endothelial cell line, MS-1, and that Rho/ROCK pathway and its downstream transcription factor myocardin-related transcription factor A (MRTF-A) positively regulated the expression of some of mesenchymal genes [34]. In order to investigate the roles of Rho/ROCK/MRTF-A axis in TGF-β-induced EndMT of HDLECs, we first examined whether Rho/ROCK pathway is involved in the EndMT. When HDLECs were treated with TGF-β2 alone or in combination with a ROCK inhibitor Y27632 for 72 h, TGF-β-induced expression of TMEPAI was significantly blocked by Y27632 (Fig 5A), suggesting Rho/ROCK pathway to be involved in transmitting TGF-β signals. Next, we examined the effect of Y27632 on the expression of lymphatic endothelial and mesenchymal cell markers. TGF-β-induced decrease in the expression of Prox1 was not altered by Y27632 treatment (Fig 5B). The presence of Y27632 also had little effect on the changes in the expression of LYVE1 induced by TGF-β (Fig 5C). On the other hand, TGF-β-induced increase in the expression of SM22α was suppressed by Y27632 (Fig 5D) but such inhibitory effect on the expression of FN1 was not observed (Fig 5E), suggesting that TGF-β-induced increase in the expression of SM22α is at least regulated via Rho/ROCK pathway. Since our previous report identified MRTF-A as a downstream effector of Rho/ROCK pathway, we next examined the effect of MRTF-A knockdown on TGF-β-induced EndMT. Introduction of MRTF-A siRNAs into HDLECs decreased TGF-β-induced expression of TMEPAI (Fig 5F and 5G), suggesting that MRTF-A regulates TGF-β signals. Decrease in the expression of Prox1 and LYVE1 observed upon TGF-β treatment, was partially suppressed by MRTF-A knockdown (Fig 5H and 5I). In addition, TGF-β-induced increase in the expression of SM22α was significantly suppressed by MRTF-A knockdown (Fig 5J), however, the increase in the expression of FN1 was only partially suppressed (Fig 5K). Taken together, these data suggest that TGF-β-induced upregulation of SM22α largely depends on Rho/ROCK/MRTF-A axis, while changes in the expression of Prox1, LYVE1, and FN1 are partially regulated by this axis.

### TNF-α potentiates TGF-β-induced EndMT of HDLECs

**Fig 6:**
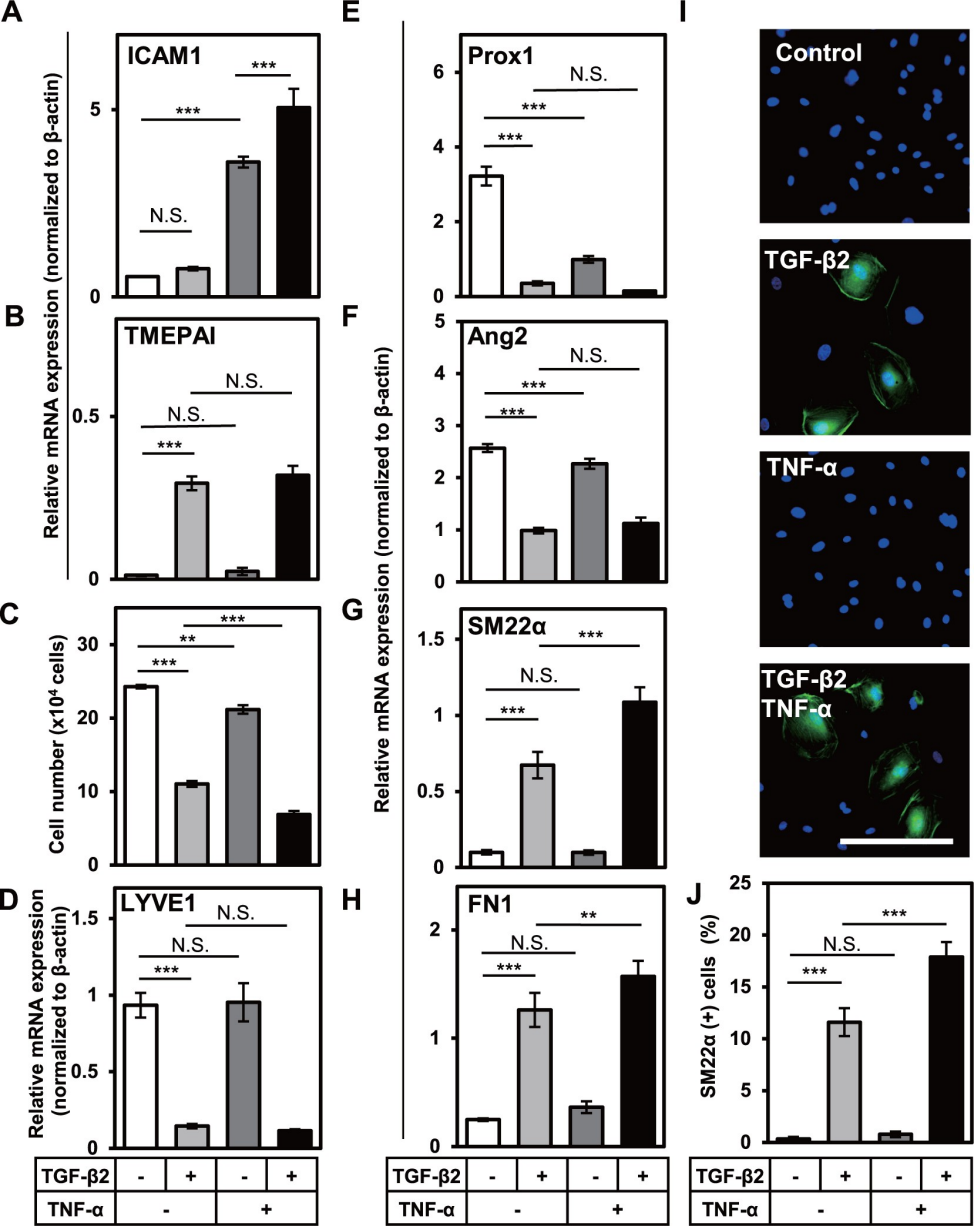
Effects of TGF-β2 and TNF-α on the cell number, and lymphatic endothelial and mesenchymal characteristics of HDLECs. HDLECs were cultured in the absence (-) or presence (+) of 0.1 or 1 ng/mL of TGF-β2 in combination with 3 or 10 ng/mL of TNF-α for 72 h, followed by qRT-PCR analysis for expression of ICAM1 (A), TMEPAI (B), LYVE1 (D), Prox1 (E), Ang2 (F), SM22α (G), and FN1 (H), by direct counting of cell numbers (C), and by fluorescence immunostaining (I) for SM22α (green) and nuclei (blue). Scale bar, 200 μm. The numbers of SM22α-positive cells were quantified (J). Data are represented as mean ± S.D., N = 4 (A, B, D-H), and as mean± S.E.M., N = 3 (C, J), representative of three independent experiments. **P < 0.01, ***P < 0.001; N.S., not significant. Differences are tested using two-way ANOVA followed by Bonferroni multiple comparison *post hoc* analysis.

We have previously reported that TNF-α, an inflammatory cytokine, enhances TGF-β-induced EMT in A549 lung adenocarcinoma cells [18]. In addition, multiple groups including ours have reported that TNF-α stimulates EndMT of vascular endothelial cells [21, 35–37]. In order to investigate the effect of TNF-α on TGF-β-induced EndMT in LECs, we first examined whether TNF-α signals are transmitted in HDLECs. When HDLECs were treated with TNF-α for 4 h, expression of ICAM1, a target gene for TNF-α signal, was significantly elevated (Fig 6A), suggesting that HDLECs are capable of transducing TNF-α signals. Next, we checked the effect of TGF-β2 on TNF-α signal by culturing HDLECs with TGF-β2 alone or in combination with TNF-α. While TNF-α did not enhance TGF-β2-induced TMEPAI expression (Fig 6B), TGF-β2 significantly increased TNF-α-induced ICAM1 expression (Fig 6A). These results suggest that TNF-α signal is transmitted in HDLECs, and TGF-β enhances TNF-α-mediated intracellular signals.

Then, we looked into the effect of TNF-α on the phenotype of HDLECs. In agreement with previous report [13], proliferation of HDLECs was decreased in the presence of TGF-β2 (Fig 6C). Although TNF-α alone had slightly suppressive effect on the growth of HDLECs, it appeared to potentiate TGF-β2-induced growth inhibition, since the treatment with a combination of both cytokines significantly decreased cell number when compared to treatment with TGF-β2 alone (Fig 6C). Similar results were also obtained using HMVEC-LLy (S1A Fig).

We next questioned whether TNF-α could induce EndMT of HDLECs. We performed qRT-PCR analysis for the expression of EndMT marker genes in HDLECs cultured for 72 h in the presence of TGF-β2, TNF-α or combination of both cytokines. The expression of a LEC marker, LYVE1, was not affected by treatment with TNF-α alone (Fig 6D). However, we could observe the decrease in the expression of other LEC markers, Prox1 and Ang2 upon a treatment with TNF-α alone (Fig 6E and 6F). On the other hand, although TNF-α alone did not affect the expression of SM22α (Fig 6G) and only slightly altered that of FN1 (Fig 6H), the expression of both mesenchymal cell markers was much higher for TGF-β2/TNF-α-treated cells compared to TGF-β2 only-treated cells (Fig 6G and 6H). These results in HDLECs were also recapitulated using HMVEC-LLy (S1B–S1F Fig).

We also analyzed the number of HDLECs exhibiting mesenchymal phenotype by counting SM22α-positive cells (Fig 6I and 6J). Although none of the control cells were SM22α-positive, more than 10% of TGF-β2-treated cells could be stained with anti-SM22α antibody. Moreover, increase in SM22α-positive cells was observed upon the combined TGF-β2/TNF-α treatment. Taken together, these data suggest that TNF-α enhances TGF-β2-induced EndMT of HDLECs.

### Signals mediated by TGF-β and TNF-α orchestrate the EndMT of LECs by regulating the expression of transcription factors

**Fig 7:**
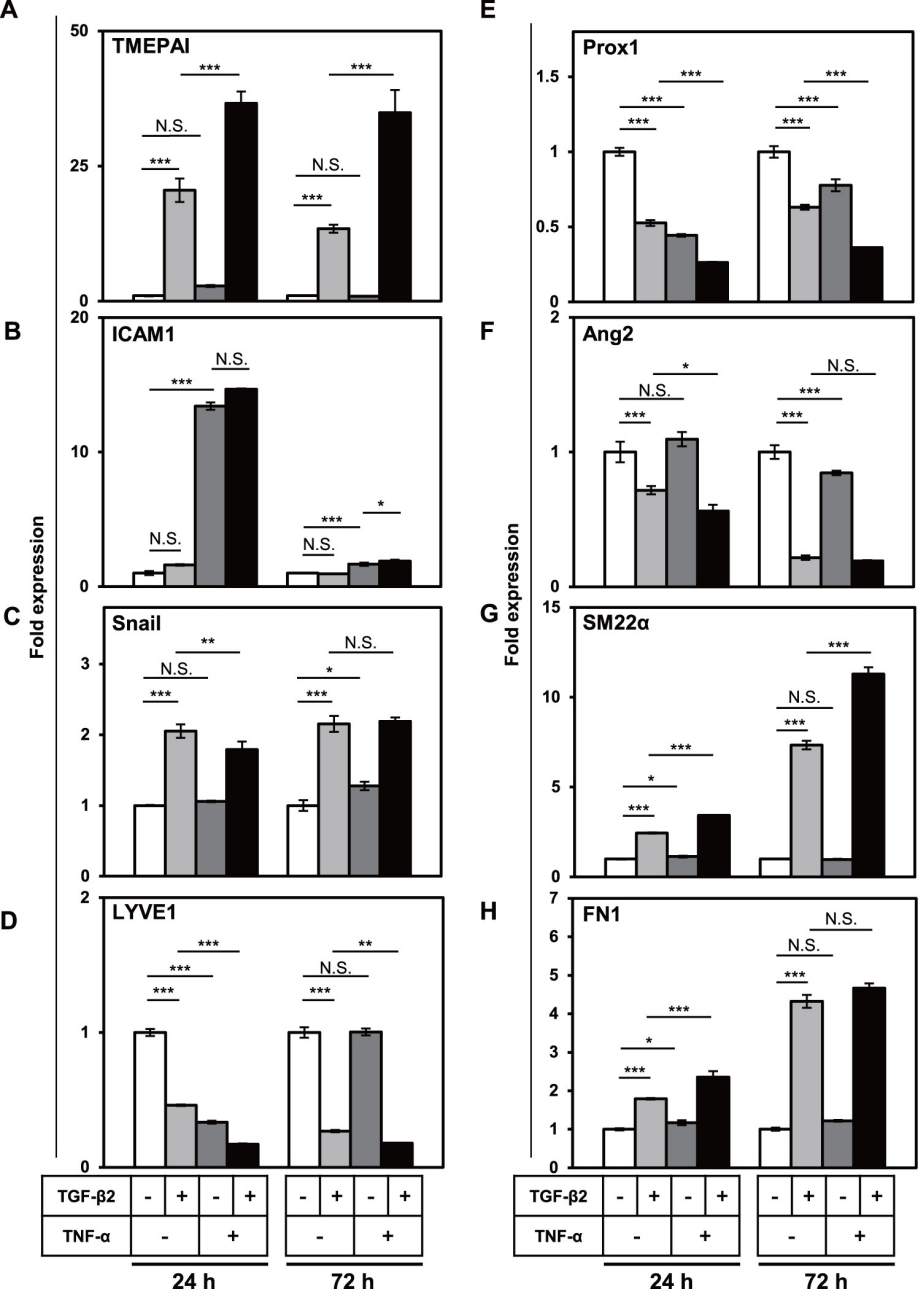
Kinetic study of the induction of various lymphatic endothelial and mesenchymal markers by TGF-β2 and TNF-α in HDLECs. HDLECs were cultured in the absence (-) or presence (+) of 0.1 ng/mL of TGF-β2 in combination with 10 ng/mL of TNF-α for the indicated periods (24 and 72 h), followed by qRT-PCR analyses for the expression of TMEPAI (A), ICAM1 (B), Snail (C), LYVE1 (D), Prox1(E), Ang2 (F), SM22α (G) and FN1 (H). Data are represented as mean ± S.D., N = 4, representative of three independent experiments. *P < 0.05, **P < 0.01, ***P < 0.001; N.S., not significant. Differences are tested using two-way ANOVA followed by Bonferroni multiple comparison *post hoc* analysis.

We have previously reported that during the EndMT of embryonic stem cell-derived endothelial cells, TGF-β2 induces the expression of Snail transcription factor, which results in the expression of smooth muscle α-actin (α-SMA), a mesenchymal marker [38]. In order to examine the causal relationship between the expression of various transcription factors regulated by both TGF-β and TNF-α and the expression of EndMT markers, we performed the kinetic study of the expression of a number of transcription factors as well as EndMT markers in TGF-β2-treated HDLECs. The expression of TMEPAI (Fig 7A) and ICAM1 (Fig 7B), direct target genes of TGF-β and TNF-α, respectively, was upregulated within 24 h after stimulation with both cytokines. It is of note that the TGF-β2-induced TMEPAI expression in HDLECs was maintained for 72 h after stimulation with TGF-β2 (Fig 7A), while TNF-α-induced ICAM1 expression was attenuated 72 h after TNF-α treatment (Fig 7B). We have also found that expression of Snail was significantly induced by TGF-β2 within 24 h after stimulation and maintained for 72 h (Fig 7C).

Our data showed that simultaneous treatment of HDLECs with TGF-β and TNF-α decreased the expression of LYVE1 (Fig 7D) and Prox1 (Fig 7E) within 24 h after stimulation, suggesting them to represent pool of direct target and early response genes. In contrast, the downregulation of Ang2 (Fig 7F) and the upregulation of SM22α (Fig 7G) and FN1 (Fig 7H) could be observed after the onset of early response genes including TMEPAI, ICAM1, Prox1, and LYVE1, and continued to increase 72 h after the stimulation, suggesting that expression of these mesenchymal markers is regulated by the transcription factors such as Snail and Prox1 that are directly controlled by TGF-β and TNF-α.

### TGF-β2-induced EndMT increases the production of endogenous Activin A by HDLECs, which leads to the sustained activation of Smad2/3 signals

**Fig 8:**
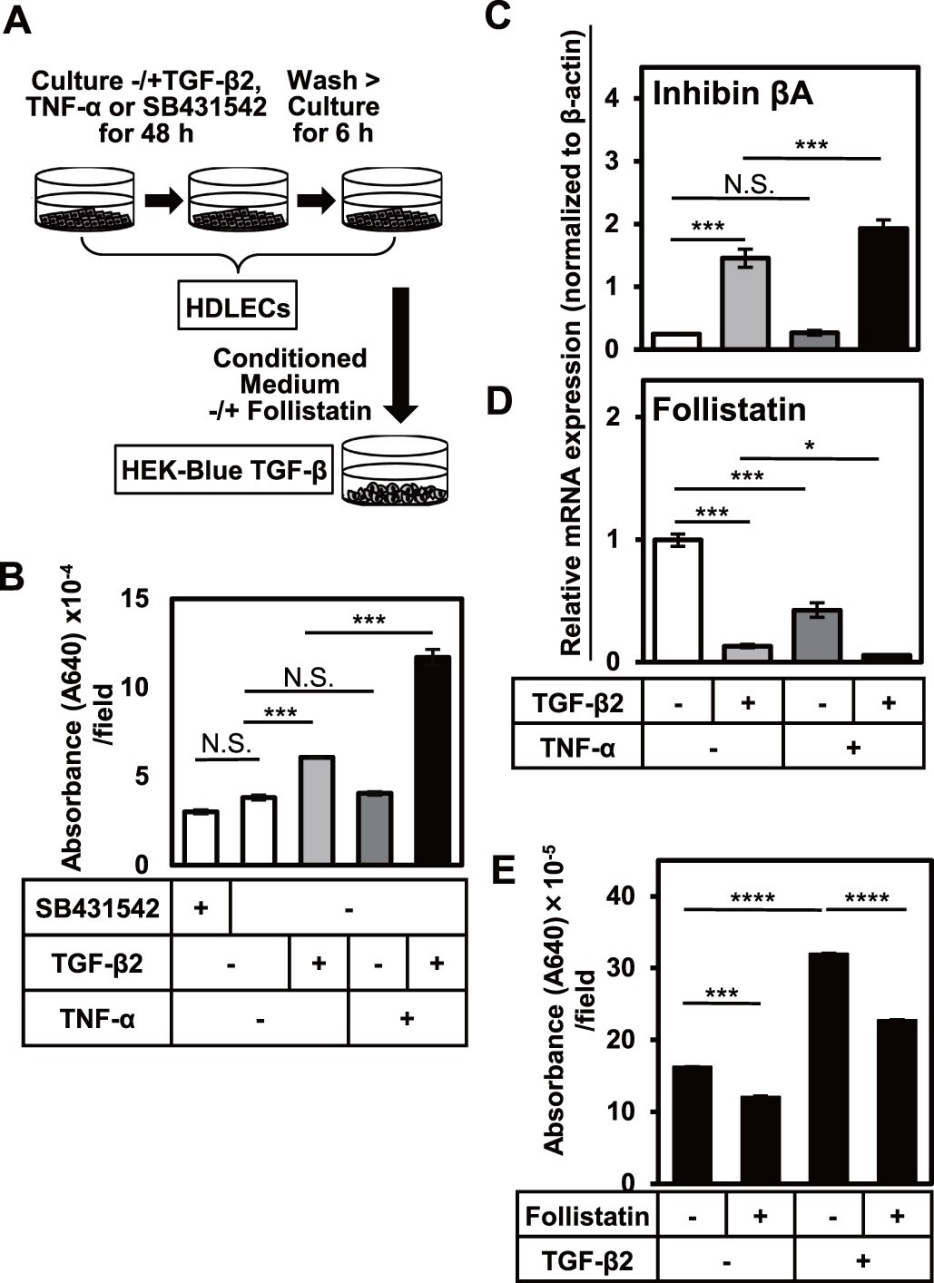
Roles of TGF-β2 and TNF-α in the production of Activin and Follistatin in HDLECs. (A, B, E) Quantification of Smad2/3-activating factors in conditioned medium of HDLECs using HEK-Blue TGF-β cells. (A) HDLECs were cultured in the absence (-) or presence (+) of 5 μM of SB431542, 0.1 ng/mL (B) or 1 ng/mL (E) of TGF-β2 in combination with 10 ng/mL of TNF-α for 48 h, followed by replacing the medium with a serum-free medium and further culture for 6 h. (B, E) HEK-Blue TGF-β cells were cultured in the conditioned medium derived from HDLECs, in the absence (-) or presence (+) of Follistatin, followed by the measurement of the absorbance at 640 nm representing colorimetric change of HEK-Blue substrate by SEAP alkaline phosphatase induced by Smad2/3 signals. (C, D) HDLECs were cultured in the absence (-) or presence (+) of 0.1 ng/mL of TGF-β2 in combination with 10 ng/mL of TNF-α for 72 h, followed by qRT-PCR analyses for the expression of Inhibin βA (C) and Follistatin (D). Values were normalized to the number of HDLECs responsible for secretion of Smad2/3-activating humoral factors. Data are represented as mean ± S.E.M., N = 3 (B, E), and as mean± S.D., N = 4 (C, D), representative of three independent experiments. *P < 0.05, ***P < 0.001, ****P < 0.0001; N.S., not significant. Differences are tested using two-way ANOVA followed by Bonferroni multiple comparison *post hoc* analysis.

Based on the present findings that the TGF-β2-induced TMEPAI expression in HDLECs was maintained 72 h after stimulation with TGF-β2 (Fig 7A), we hypothesized that EndMT induces the production of “humoral factor activating Smad2/3 signal” (Smad2/3 activating factor) from LECs. In order to test this hypothesis, we collected conditioned medium of HDLECs cultured in a presence of SB431542, TGF-β2 or TNF-α for 48 h and analyzed it using TGF-β signal-responsive HEK-Blue reporter cell system (Fig 8A). The HEK-Blue TGF-β cells were cultured for 24 h and the activity of “Smad2/3 activating humoral factor” produced by HDLECs was examined. In the conditioned medium of HDLEC supplemented with TGF-β2, activation of TGF-β signal was enhanced by “Smad2/3 activating humoral factor” if compared to the untreated control (Fig 8B). This effect was even more enhanced by combined treatment with TGF-β2 and TNF-α (Fig 8B). These results suggest that exogenous TGF-β2 and TNF-α in HDLECs induce EndMT by activating Smad pathway and increasing secretion of a “Smad2/3 activating humoral factor”, suggesting EndMT to be further enhanced in an exogenous TGF-β2-independent manner.

In order to identify the “Smad2/3 activating humoral factor” whose expression was increased in HDLECs upon stimulation with TGF-β2, we performed the expression analysis of the TGF-β superfamily members. At first, we focused on the expression of TGF-β1, TGF-β2, and TGF-β3, prototypes of the TGF-β family. There was no significant upregulation in the expression of any of prototypes of the TGF-β family by the addition of exogenous TGF-β2 (S4 Fig). Thus, in the next step we examined the expression of subunits of Nodal and Activin, other members of TGF-β superfamily. Only Inhibin βA, the subunit of Activin A, was expressed in HDLECs (Fig 8C) among all ligands studied, and we found that the TGF-β2 treatment induced the expression of Inhibin βA, which was enhanced by a combined treatment with TNF-α and TGF-β2. Moreover, the expression of Follistatin, an inhibitor of Activin, decreased in HDLECs cultured in a presence of TGF-β2 (Fig 8D), leading us to the conclusion that upon TGF-β2 treatment HDLECs secrete Activin A and decrease the expression of Follistatin, that results in TGF-β-independent phosphorylation of Smad2 and enhanced EndMT.

We further examined the physiological roles of Activin A produced by HDLECs in the activation of Smad2/3 signals by using HEK-Blue reporter cells. When the conditioned medium from HDLECs was added to HEK-Blue cells in combination with Follistatin, the increased activation of Smad2/3 signals seen upon incubation with the conditioned medium was partially decreased, suggesting endogenous Activin A to be responsible for the activation of Smad2/3 signals (Fig 8E). These results suggest that TGF-β2 induced EndMT increased the production of endogenous Activin A from HDLECs, which led to the sustained activation of Smad2/3 signals.

The clarified roles of Activin A, which is induced by TGF-β2 treatment, further prompted us to examine the effects of simultaneous stimulation of HDLECs with Activin A and TGF-β2. The qRT-PCR analyses of the expression of a TGF-β target gene, TMEPAI (S5A Fig), and EndMT markers, LYVE1, Prox1, SM22α, and FN1 (S5B–S5E Fig), revealed that Activin A has an additive effect on regulating expression of these genes, suggesting Activin A and TGF-β2 transduce their signals, via different receptors; possibly ALK4 and ALK5, respectively. The effect seemed to be limited, compared to that of TNF-α, probably because both Activin A and TGF-β2 transduce their signals via Smad2/3.

We next examined the physiological roles of the endogenous expression of Follistatin since we could observe significant downregulation of Follistatin by TGF-β and TNF-α (Fig 8D). When Follistatin was knocked down using siRNA, the expression of lymphatic endothelial markers, LYVE1 and Prox1, was significantly decreased (S5F–S5H Fig). At the same time, we could also observe strong upregulation in SM22α expression and only slight increase in FN1 expression (S5I and S5J Fig). These results suggest that high expression of Follistatin is maintained in physiological state of HDLECs in order to regulate the endogenous level of Activin A to suppress EndMT. Our data also revealed that Activin A, whose expression is elevated by exogenous TGF-βs, is an endogenous factor that stabilizes the EndMT of LECs.

### Human dermal lymphatic vessels undergo mesenchymal transition during senescence

Previous reports suggested that signals mediated by TGF-β and TNF-α increased during senescence [39]. Glaucoma is one of the well-known senescence-associated diseases. One of the causes of glaucoma is the dysfunction of Schlemm’s canal (SC) endothelial cells whose property resembles lymphatic endothelial cells due to Prox1 expression. Expression of TGF-β2 has been reported to be upregulated in SC endothelial cells isolated from glaucoma patients [40]. Furthermore, EndMT-like phenomenon was observed in SC in concomitant with decreased expression of Prox1 [41]. Since it has been reported that the expression of TGF-β2 in SC is higher in glaucoma patients if compared with healthy individuals [40], we examined the expression of TGF-β2 in various tissues of aged mice. As shown in S6 Fig, expression of TGF-β2 in ear and abdominal skin tissues was significantly higher in ear and abdominal skin tissues of old mice compared to young mice, further suggesting TGF-β2 to be one of the causal genes for EndMT of dermal LECs in mice during aging.

**Fig 9:**
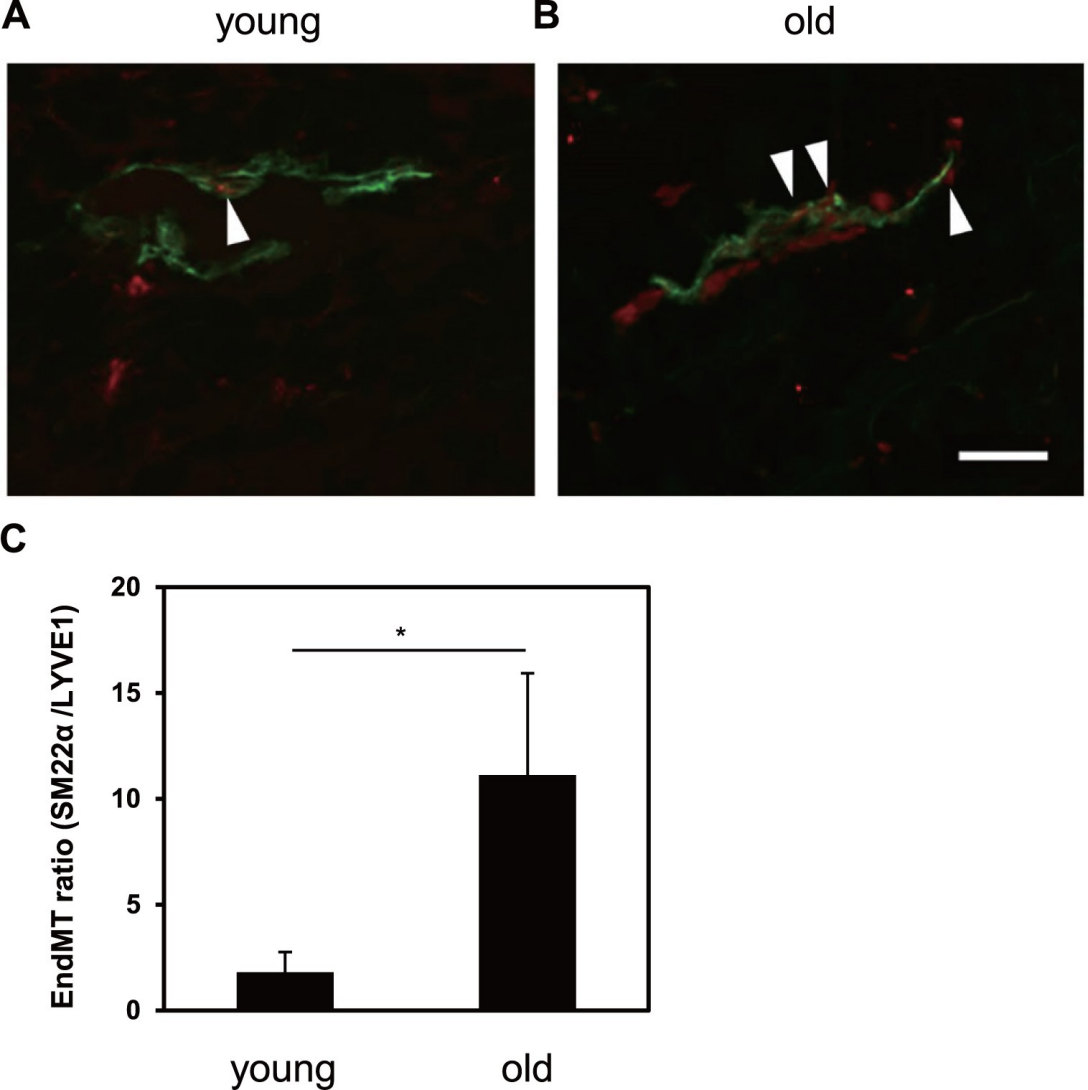
Alterations of lymphatic vasculature in aged human skin. (A, B) Sections of skin tissues from young and old volunteers were subjected to immunofluorescence staining for LYVE1 (green) and SM22α (red). Representative images of immunostained samples of young (A) and old volunteers (B). Arrowheads indicate EndMT cells co-expressing LYVE1 and SM22α. Scale bar, 100 μm. (C) Levels of EndMT ratio were quantified as described in Materials and Methods. Data are represented as mean ± S.E.M., N = 8 (young age) and N = 10 (old age), representative of two independent experiments. *P < 0.05. Differences are tested using Student's *t*-test.

Next, in order to confirm the physiological relevance of our *in vitro* findings that TGF-β and TNF-α induce EndMT, we performed double immunostaining for LYVE1 and SM22α using young and aged human dermal tissues to assess the level of EndMT changes during aging. Fluorescence microscopy analyses revealed that the total area covered by LYVE1^+^ vessels was decreased in the aged skin samples (Fig 9A and 9B). Furthermore, the area covered by SM22α^+^ LYVE1^+^ vessels in the eyelid skin was increased in the aged skin samples. We quantitatively measured the EndMT ratio by calculating the ratio of the total area of SM22α^+^ LYVE1^+^ lymphatic vessels to that of LYVE1^+^ lymphatic vessels per area unit (mm^2^). This analysis confirmed that the density of SM22α^+^ LYVE1^+^ lymphatic vessels was significantly increased in aged skin (Fig 9C). These results suggest that human dermal lymphatic vessels undergo EndMT during aging which eventually leads to a reduction of lymphatic vessels.

## Discussion

**Fig 10:**
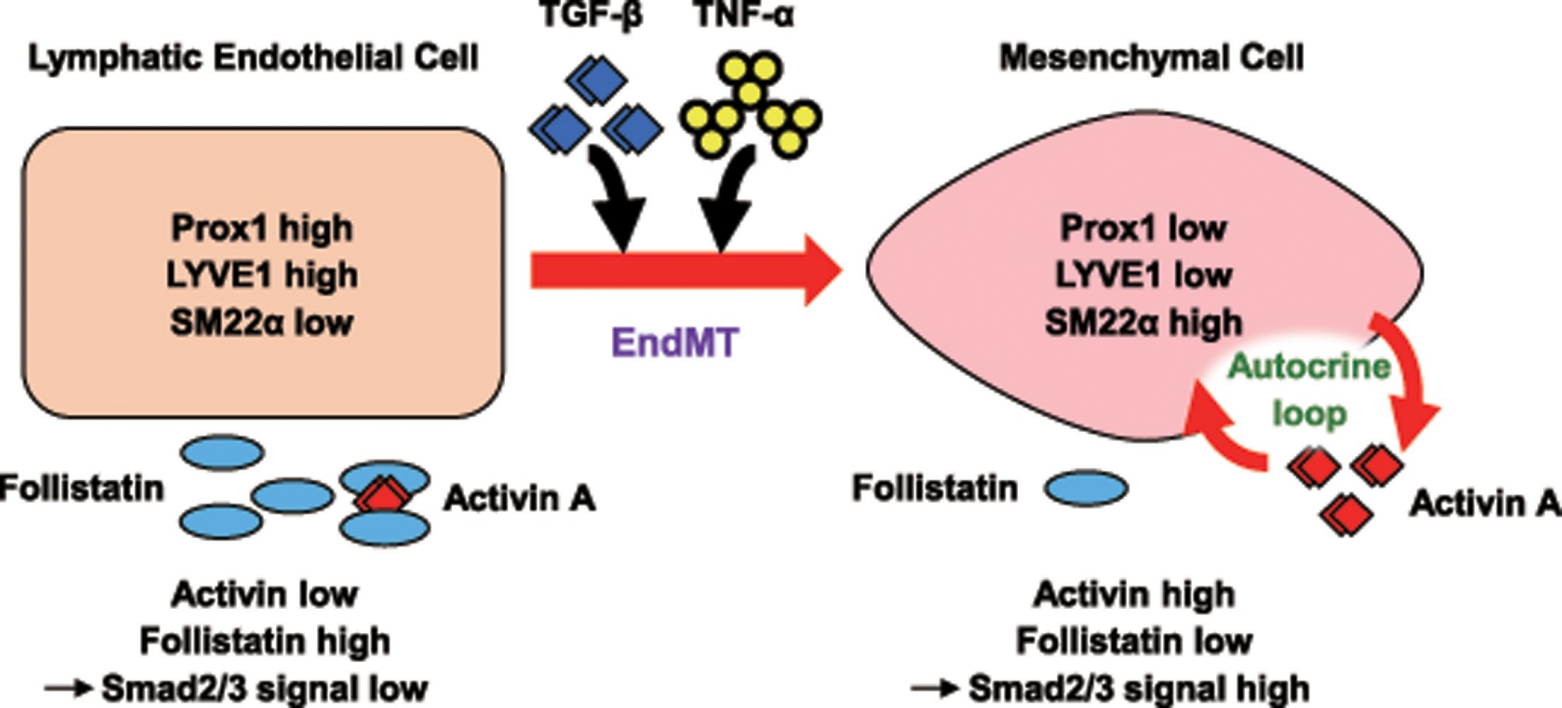
Schematic diagram of the proposed mechanism for the stabilization of EndMT of lymphatic endothelial cells triggered by TGF-β and TNF-α. In lymphatic endothelial cells (LECs), expression of LEC markers, Prox1 and LYVE1, is maintained due to the low level of Smad2/3 signals that are activated by TGF-βs and Activins. TGF-β and TNF-α induces EndMT, which is characterized by decreased expression of LEC markers and increased expression of mesenchymal markers including SM22α. During EndMT of LECs, production of Activin A is increased while the expression of its inhibitory molecule Follistatin is decreased, which leads to activation of autocrine loop of Activin signals. Such changes result in the activation of Smad2/3 signal in LEC-derived mesenchymal cells and stabilization of EndMT.

In the present study, we revealed for the first time that EndMT of LECs is induced by TGF-β2 and further enhanced by TNF-α. Furthermore, we found that TGF-β-induced activation of the autocrine Activin signal loop, likely assures the irreversible and long-lasting effect induced by TGF-β2 and TNF-α (Fig 10).

We previously reported that transcriptional changes of mesenchymal markers during EndMT of BECs were controlled by Snail and MRTF-A transcription factors whose expression increased in response to TGF-β signals [38, 34]. In the present study, we observed that TGF-β2 induced the expression of Snail (Fig 7C) in HDLECs. In addition, our data suggested that MRTF-A is a key transcription factor that regulates the expression of mesenchymal markers such as SM22αand at least partially affects the expression of LEC markers (Fig 5). Putative upstream Rho/ROCK signals were also shown to regulate the expression of lymphatic endothelial cell and mesenchymal markers. Furthermore, we found that TGF-β2 decreased the expression of Prox1, a transcription factor which is indispensable for maintaining the phenotypes of adult LECs [15]. Prox1 plays these roles by inducing the expression of various LEC markers, including Ang2. Thus, decreased expression of Prox1 in response to TGF-β2 would likely affect the expression level of the genes responsible for maintaining the properties of the LECs, such as Ang2, leading to the loss of LEC identity.

Our previous reports showed that BMP-9, a member of the TGF-β family, decreases the number of HDLECs by lowering Proxl expression [14]. BMP-9 binds to the receptor complexes comprising of ALK1 type I receptor, activates Smad1, 5, and 8, and by this means regulates the expression of a group of target genes different from the ones activated by TGF-β/ALK5/Smad2, 3 signaling pathway. While BMP-9 and TGF-β transduce their signals via different Smad complex, considering loss of LEC phenotype in a presence of TGF-β2 as well as its anti-proliferative effect on LECs, it is likely that TGF-β2 suppresses lymphangiogenesis in the same or a very similar manner as BMP-9 (S2 Fig) [13, 14].

We found that expression of TMEPAI, a direct target of Smad2/3 signals, was upregulated at 24 h after TGF-β stimulation and maintained even at 72 h post-stimulation (Fig 7A). The present study revealed that this sustained activation of Smad2/3 signals was mediated by the activation of an autocrine Activin signal loop, caused by increased production of Activin A and decreased production of Follistatin by TGF-β2-treated HDLECs (Fig 8). These results suggest that Smad2/3 signals, which are triggered by exogenous TGF-β, do not require additional TGF-β ligands once autocrine Activin signals are activated (Fig 10). Although this mechanism is beneficial in stabilizing EndMT, it is also disadvantageous from the point of view of maintaining the properties of LECs. Various signals and transcription factors have been reported to suppress the induction of TGF-β-dependent EndMT. Ichise and colleagues reported that MEK, which is known to be activated in LECs by FGF signal, phosphorylates the linker site of Smad2, thereby inhibiting TGF-β signal and suppressing EndMT [17]. Several other groups showed that in BECs, FGF signal can suppress EndMT induced by TGF-β [42]. While LECs may also have a network of signal transcription factors that would oppose the induction of TGF-β-dependent EndMT, elucidation of detailed molecular mechanisms requires further studies.

Neo-lymphangiogenesis has been observed in various tissues in which inflammation takes place [43]. Various inflammatory cells such as macrophages accumulating at inflammatory sites produce vascular endothelial growth factor (VEGF)-A and VEGF-D and thus stimulate endothelial cell proliferation and movement in existing lymphatic vessels. TNF-α is one of the pro-inflammatory cytokines that has been reported to contribute to an increased vascular permeability seen during inflammation. Flister and colleagues reported that inflammatory cytokine IL-3 activates NF-κB pathway which results in stimulation of expression of VEGF receptor 3 through the elevated Prox1 expression in HDLECs [44]. Our present data revealed that the inflammatory cytokine TNF-α cooperates with TGF-β in decreasing the expression of Prox1. It is known that in addition to NF-κB pathway, TNF-α can enhance cell motility by activating various intracellular signaling pathways [20], thus decreased expression of Prox1 in a presence of TNF-α may result from the activation of pathways independent of NF-κB. We have previously reported that combined treatment with TNF-α and TGF-β induces EMT of lung cancer cells [18]. These findings suggest that TGF-β and TNF-α cooperate to induce mesenchymal transition of both epithelial and endothelial cells via similar mechanisms.

It has been proposed that inflammation is enhanced with aging [24]. Recent lines of evidence suggest that lymphatic dysfunction associated with aging leads to a variety of pathological conditions. As described above, several groups have reported that the senescence-associated development of glaucoma is caused by functional deterioration of SC [45]. It has been reported that EndMT of SC endothelial cells that occurs during aging, plays an important role in the malfunction of SC and the development of glaucoma [41]. We showed that the expression of TGF-β2 was elevated in dermal tissues of aged mice (S6 Fig). Taken together with a previous report that the expression of TGF-β2 in SC is higher in glaucoma patients if compared with healthy individuals [40], it is possible that EndMT may be induced by the senescence-associated increased expression of TGF-β2 in SC or its surroundings. Alzheimer’s disease is also an age-related disease. Da Mesquita and colleagues reported that dysfunction of the lymphatic vessels of the central nervous dura is a main factor in the onset of Alzheimer's disease [46].

Some reports showed that lymphedema caused by a dysfunction of lymphatic vessels in lymphedema model mice can be improved by blocking of TGF-β1 [47]. Therefore, it is likely that TGF-β is involved in a formation of lymphedema, by stimulating EndMT of LECs [17]. In this study, we have found that dermal lymphatic endothelial cells in older human subjects expressed a mesenchymal marker, SM22α, at a significantly higher rate, compared to younger counterparts, suggesting that EndMT in human dermis can be induced with age. Whether inflammation signals in addition to TGF-β contribute to enhancement of EndMT in LECs residing in dermal tissues needs to be elucidated in the future. Taken together with the present findings, EndMT induced by TGF-β is expected to be a good target for prevention and treatment of lymphatic dysfunction-related pathological conditions.

## Supporting information

S1 Text (DOCX)

S1 Fig Effects of TGF-β2 and TNF-α on the cell number, and lymphatic endothelial and mesenchymal characteristics of HMVEC-LLy. HDLECs were cultured in the absence (-) or presence (+) of 1 ng/mL of TGF-β2 in combination with 10 ng/mL of TNF-α for 72 h, followed by direct counting of cell number (A) and qRT-PCR analyses for the expression of LYVE1 (B), Prox1 (C), Ang2 (D), SM22α (E) and FN1 (F). Data are represented as mean± S.D., N = 4 (B-F), and as mean± S.E.M., N = 3 (A), representative of three independent experiments. *P < 0.05, **P < 0.01, ***P < 0.001; N.S., not significant. Differences are tested using two-way ANOVA followed by Bonferroni multiple comparison *post hoc* analysis.
(TIF)

S2 Fig Effects of TGF-β2 and BMP-9 on lymphatic endothelial and mesenchymal characteristics of HDLECs. (A-C, E-G) HDLECs were cultured in the absence (-) or presence (+) of 1 ng/mL of TGF-β2 in combination with 2 ng/mL of BMP-9 for 24 h, followed by qRT-PCR analyses for the expression of ID1 (A), TMEPAI (B), LYVE1 (C), FN1 (E), Ang2 (F) and SM22α (G). (D) HDLECs were cultured in the absence (-) or presence (+) of 0.1 ng/mL of TGF-β2 in combination with 1 ng/mL of BMP-9 for 4 h, followed by qRT-PCR analysis for the expression of Prox1. Data are represented as mean± S.D., N = 4, representative of three independent experiments. *P < 0.05, **P < 0.01, ***P < 0.001; N.S., not significant. Differences are tested using two-way ANOVA followed by Bonferroni multiple comparison *post hoc* analysis.
(TIF)

S3 Fig Roles of Smad4 in the TGF-β-induced change in lymphatic endothelial and mesenchymal characteristics of HDLECs. HDLECs transfected with negative control siRNA (NC) or siRNAs for Smad4 (Smad4-A and Smad4-B) were cultured in the absence (-) or presence (+) of 1.0 ng/ml of TGF-β2 for 72 h, followed by qRT-PCR analyses for the expression of Smad4 (A), TMEPAI (B), LYVE1 (C), and SM22α (D). Data are represented as mean± S.D., N = 4, representative of three independent experiments. ***P < 0.001. Differences are tested using one-way ANOVA (A) or two-way ANOVA (B-D) followed by Bonferroni multiple comparison *post hoc* analysis.
(TIF)

S4 Fig Effects of TGF-β2 on the expression of TGF-β family ligands. HDLECs were cultured in the absence (-) or presence (+) of 0.1 ng/mL of TGF-β 2 in combination with 10 ng/mL of TNF-α for 72 h, followed by qRT-PCR analyses for the expression of TGF-β 1 (A), TGF-β 2 (B), and TGF-β 3 (C). Data are represented as mean± S.D., N = 4, representative of three independent experiments. ***P < 0.001; N.S., not significant. Differences are tested using two-way ANOVA followed by Bonferroni multiple comparison *post hoc* analysis.
(TIF)

S5 Fig Effects of TGF-β 2, Activin A and Follistatin on the lymphatic endothelial and mesenchymal characteristics of HDLECs. (A-E) HDLECs were cultured in the absence (-) or presence (+) of 0.1 ng/mL of TGF-β 2 in combination with 10 ng/mL of Activin A for 72 h, followed by qRT-PCR analyses for the expression of TMEPAI (A), LYVE1 (B), Prox1 (C), SM22α (D), and FN1 (E). (F-J) HDLECs transfected with negative control siRNA (NC) or siRNAs for Follistatin (FST-A and FST-B) were cultured for 48 h, followed by qRT-PCR analyses for the expression of Follistatin (F), LYVE1 (G), Prox1 (H), SM22α (I), and FN1 (J). Data are represented as mean± S.D., N = 4, representative of three independent experiments. *P < 0.05, **P < 0.01, ***P < 0.001; N.S., not significant. Differences are tested using two-way ANOVA (A-E) or one-way ANOVA (F-J) followed by Bonferroni multiple comparison *post hoc* analysis.
(TIF)

S6 Fig Expression of TGF-β 2 in the skins of young and aged mice. Ear and abdominal skin tissues were dissected from young (2 months) and aged (14–17 months) mice followed by qRT-PCR analyses for the expression of TGF-β 2 in ear skin (A) and abdominal skin (B). Data are represented as mean±S.E.M., N = 3, representative of two independent experiments. *P < 0.05. Differences are tested using Student t-test.
(TIF)
