# Supplementary material for: TGF-beta and TNF-alpha cooperatively induce mesenchymal transition of lymphatic endothelial cells via activation of Activin signals
Source: PLoS One. 2020 May 1;15(5):e0232356. doi: 10.1371/journal.pone.0232356 (PMC7194440; doi:10.1371/journal.pone.0232356)
Supplement: S1 Text — (DOCX) [file pone.0232356.s001.docx]

**Supporting Information**

**Materials and Methods**

**Cells culture and reagents**

HMVEC-LLy (Lung LECs) were purchased from Lonza (Basel, Switzerland). The cells were maintained in EBM ^TM^-2MV Bullet Kit (cc-3202, Lonza) and used for study after extensive characterization. BMP9 was purchased from R&D systems (Minneapolis, MN, USA).

**RNA interference**

The siRNAs for human Follistatin (Cat. No.S100087745, No.S100087752) and negative control were purchased from QIAGEN (Hilden, Germany). The siRNAs for human SMAD4 (#A: 5’-GCAAAGGUGUGCAGUUGGAAUGUAA-3’, #B: 5’-GGCAAAGGUGUGCAGUUGGAAUGUA-3’) and negative control were purchased from Invitrogen.

**RNA isolation from mouse skin tissues**

Ear skin and abdominal skin from mice were dissected and minced, followed by total RNA isolation with NucleoSpin Tissue RNA extraction kit (TaKaRa Bio) according to protocol supplied by the manufacturer.

**Animal Study**

Animal experiments were approved by Institutional Animal Care and Use Committee of Tokyo Medical and Dental University (registration number: A2019-251C2) and were done according to the guidelines of the Animal Care Standards of Tokyo Medical and Dental University.

**Figure captions**

**S1 Fig. Effects of TGF-β2 and TNF-α on the cell number, and lymphatic endothelial and mesenchymal characteristics of HMVEC-LLy.** HDLECs were cultured in the absence (-) or presence (+) of 1 ng/mL of TGF-β2 in combination with 10 ng/mL of TNF-α for 72 h, followed by direct counting of cell number (A) and qRT-PCR analyses for the expression of LYVE1 (B), Prox1 (C), Ang2 (D), SM22α (E) and FN1 (F). Data are represented as mean± S.D., N=4 (B-F), and as mean± S.E.M., N=3 (A), representative of three independent experiments. *P < 0.05, **P < 0.01, ***P < 0.001; N.S., not significant. Differences are tested using two-way ANOVA followed by Bonferroni multiple comparison *post hoc* analysis.

**S2 Fig.** **Effects of TGF-β2 and BMP-9 on lymphatic endothelial and mesenchymal characteristics of HDLECs.** (A-C, E-G) HDLECs were cultured in the absence (-) or presence (+) of 1 ng/mL of TGF-β2 in combination with 2 ng/mL of BMP-9 for 24 h, followed by qRT-PCR analyses for the expression of ID1 (A), TMEPAI (B), LYVE1 (C), FN1 (E), Ang2 (F) and SM22α (G). (D) HDLECs were cultured in the absence (-) or presence (+) of 0.1 ng/mL of TGF-β2 in combination with 1 ng/mL of BMP-9 for 4 h, followed by qRT-PCR analysis for the expression of Prox1. Data are represented as mean± S.D., N=4, representative of three independent experiments. *P < 0.05, **P < 0.01, ***P < 0.001; N.S., not significant. Differences are tested using two-way ANOVA followed by Bonferroni multiple comparison *post hoc* analysis.

**S3 Fig. Roles of Smad4 in the TGF-β-induced change in lymphatic endothelial and mesenchymal characteristics of HDLECs.** HDLECs transfected with negative control siRNA (NC) or siRNAs for Smad4 (Smad4-A and Smad4-B) were cultured in the absence (-) or presence (+) of 1.0 ng/ml of TGF-β2 for 72 h, followed by qRT-PCR analyses for the expression of Smad4 (A), TMEPAI (B), LYVE1 (C), and SM22α (D). Data are represented as mean± S.D., N=4, representative of three independent experiments. ***P < 0.001. Differences are tested using one-way ANOVA (A) or two-way ANOVA (B-D) followed by Bonferroni multiple comparison *post hoc* analysis.

**S4 Fig.** **Effects of TGF-β2 on the expression of TGF-β family ligands.** HDLECs were cultured in the absence (-) or presence (+) of 0.1 ng/mL of TGF-β2 in combination with 10 ng/mL of TNF-α for 72 h, followed by qRT-PCR analyses for the expression of TGF-β1 (A), TGF-β2 (B), and TGF-β3 (C). Data are represented as mean± S.D., N=4, representative of three independent experiments. ***P < 0.001; N.S., not significant. Differences are tested using two-way ANOVA followed by Bonferroni multiple comparison *post hoc* analysis.

**S5 Fig.** **Effects of TGF-β2, Activin A and Follistatin on the lymphatic endothelial and mesenchymal characteristics of HDLECs.** (A-E) HDLECs were cultured in the absence (-) or presence (+) of 0.1 ng/mL of TGF-β2 in combination with 10 ng/mL of Activin A for 72 h, followed by qRT-PCR analyses for the expression of TMEPAI (A), LYVE1 (B), Prox1 (C), SM22α (D), and FN1 (E). (F-J) HDLECs transfected with negative control siRNA (NC) or siRNAs for Follistatin (FST-A and FST-B) were cultured for 48 h, followed by qRT-PCR analyses for the expression of Follistatin (F), LYVE1 (G), Prox1 (H), SM22α (I), and FN1 (J). Data are represented as mean± S.D., N=4, representative of three independent experiments. *P < 0.05, **P < 0.01, ***P < 0.001; N.S., not significant. Differences are tested using two-way ANOVA (A-E) or one-way ANOVA (F-J) followed by Bonferroni multiple comparison *post hoc* analysis.

**S6. Fig. Expression of TGF-β2 in the skins of young and aged mice.** Ear and abdominal skin tissues were dissected from young (2 months) and aged (14-17 months) mice followed by qRT-PCR analyses for the expression of TGF-β2 in ear skin (A) and abdominal skin (B). Data are represented as mean±S.E.M., N=3, representative of two independent experiments. *P < 0.05. Differences are tested using Student t-test.
