# Supplementary figures and images for: TGF-beta and TNF-alpha cooperatively induce mesenchymal transition of lymphatic endothelial cells via activation of Activin signals
Source: PLoS One. 2020 May 1;15(5):e0232356. doi: 10.1371/journal.pone.0232356 (PMC7194440; doi:10.1371/journal.pone.0232356)

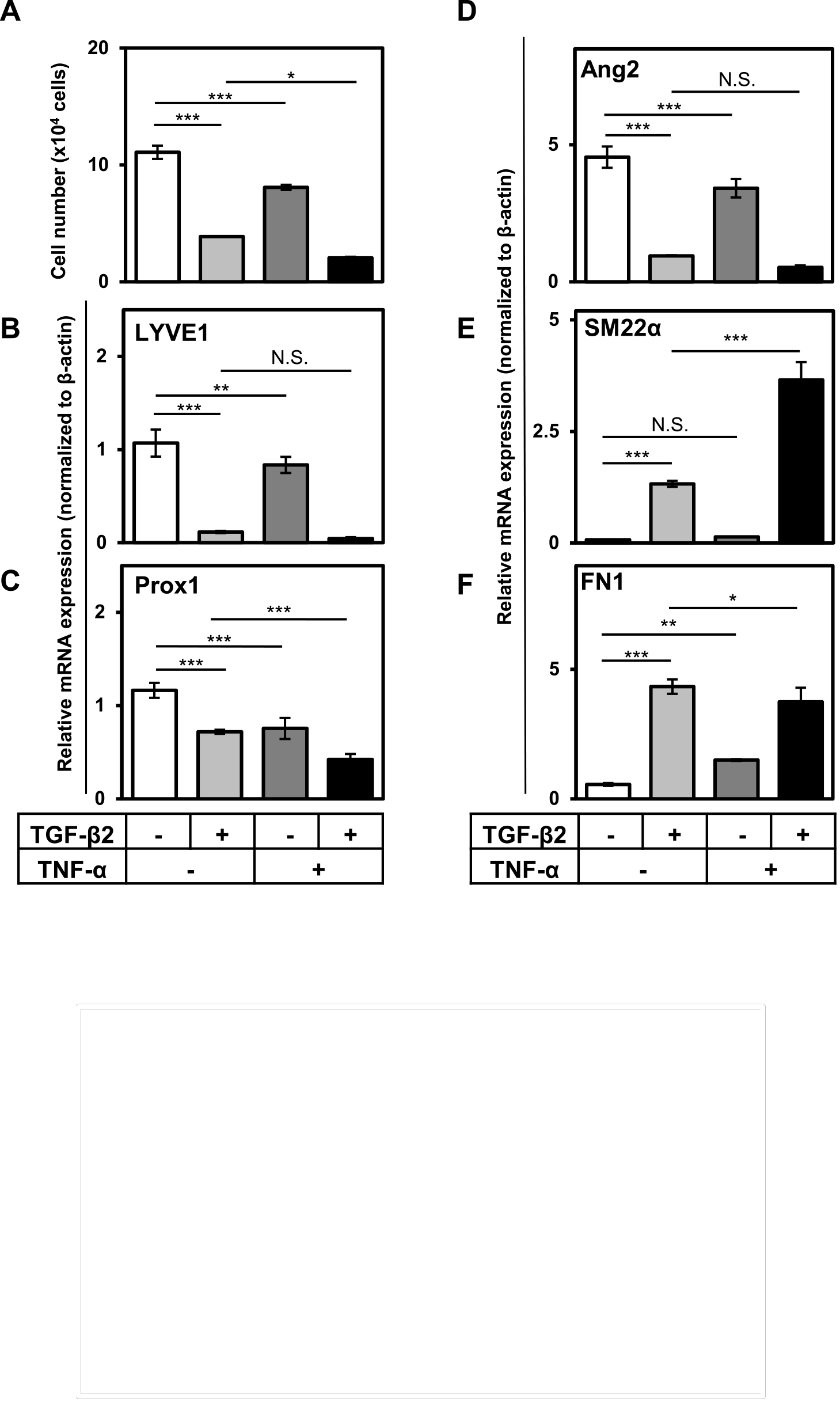

Supplement: S1 Fig — HDLECs were cultured in the absence (-) or presence (+) of 1 ng/mL of TGF-β2 in combination with 10 ng/mL of TNF-α for 72 h, followed by direct counting of cell number (A) and qRT-PCR analyses for the expression of LYVE1 (B), Prox1 (C), Ang2 (D), SM22α (E) and FN1 (F). Data are represented as mean± S.D., N = 4 (B-F), and as mean± S.E.M., N = 3 (A), representative of three independent experiments. *P < 0.05, **P < 0.01, ***P < 0.001; N.S., not significant. Differences are tested using two-way ANOVA followed by Bonferroni multiple comparison post hoc analysis. (TIF) [file pone.0232356.s002.tif]

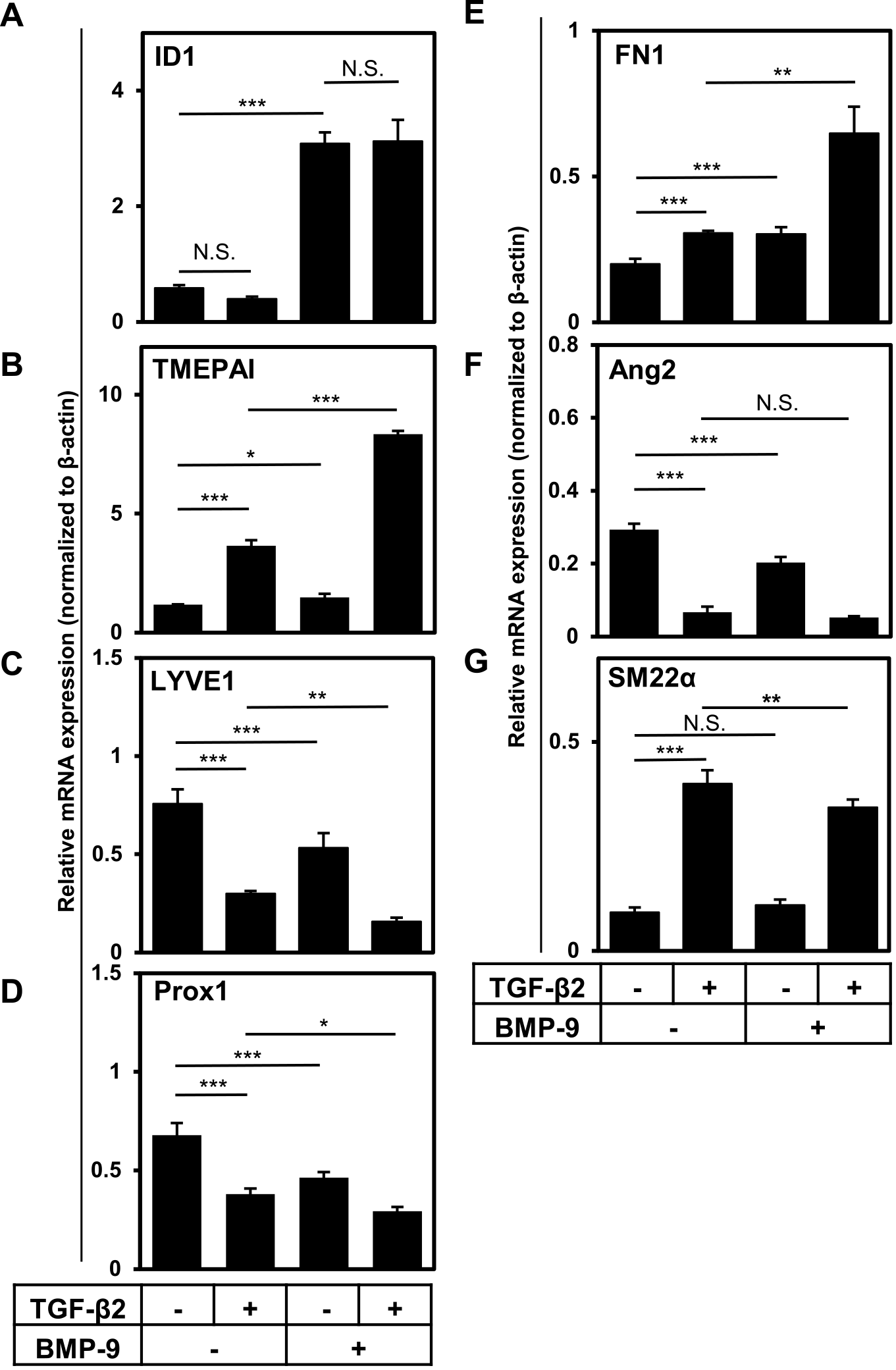

Supplement: S2 Fig — (A-C, E-G) HDLECs were cultured in the absence (-) or presence (+) of 1 ng/mL of TGF-β2 in combination with 2 ng/mL of BMP-9 for 24 h, followed by qRT-PCR analyses for the expression of ID1 (A), TMEPAI (B), LYVE1 (C), FN1 (E), Ang2 (F) and SM22α (G). (D) HDLECs were cultured in the absence (-) or presence (+) of 0.1 ng/mL of TGF-β2 in combination with 1 ng/mL of BMP-9 for 4 h, followed by qRT-PCR analysis for the expression of Prox1. Data are represented as mean± S.D., N = 4, representative of three independent experiments. *P < 0.05, **P < 0.01, ***P < 0.001; N.S., not significant. Differences are tested using two-way ANOVA followed by Bonferroni multiple comparison post hoc analysis. (TIF) [file pone.0232356.s003.tif]

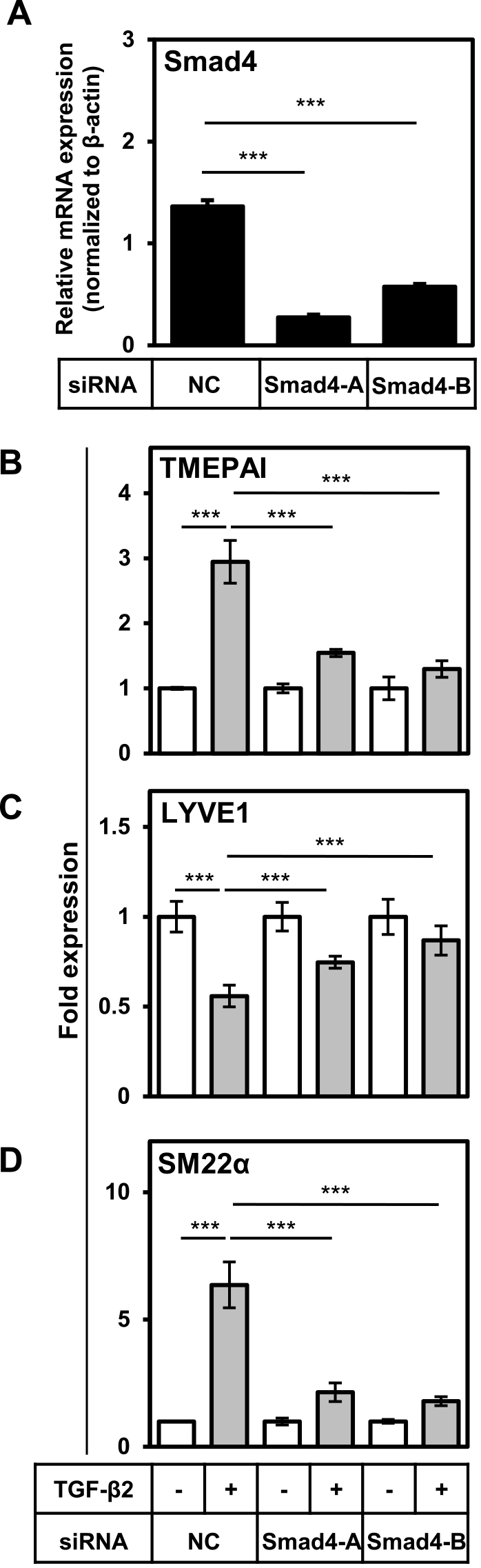

Supplement: S3 Fig — HDLECs transfected with negative control siRNA (NC) or siRNAs for Smad4 (Smad4-A and Smad4-B) were cultured in the absence (-) or presence (+) of 1.0 ng/ml of TGF-β2 for 72 h, followed by qRT-PCR analyses for the expression of Smad4 (A), TMEPAI (B), LYVE1 (C), and SM22α (D). Data are represented as mean± S.D., N = 4, representative of three independent experiments. ***P < 0.001. Differences are tested using one-way ANOVA (A) or two-way ANOVA (B-D) followed by Bonferroni multiple comparison post hoc analysis. (TIF) [file pone.0232356.s004.tif]

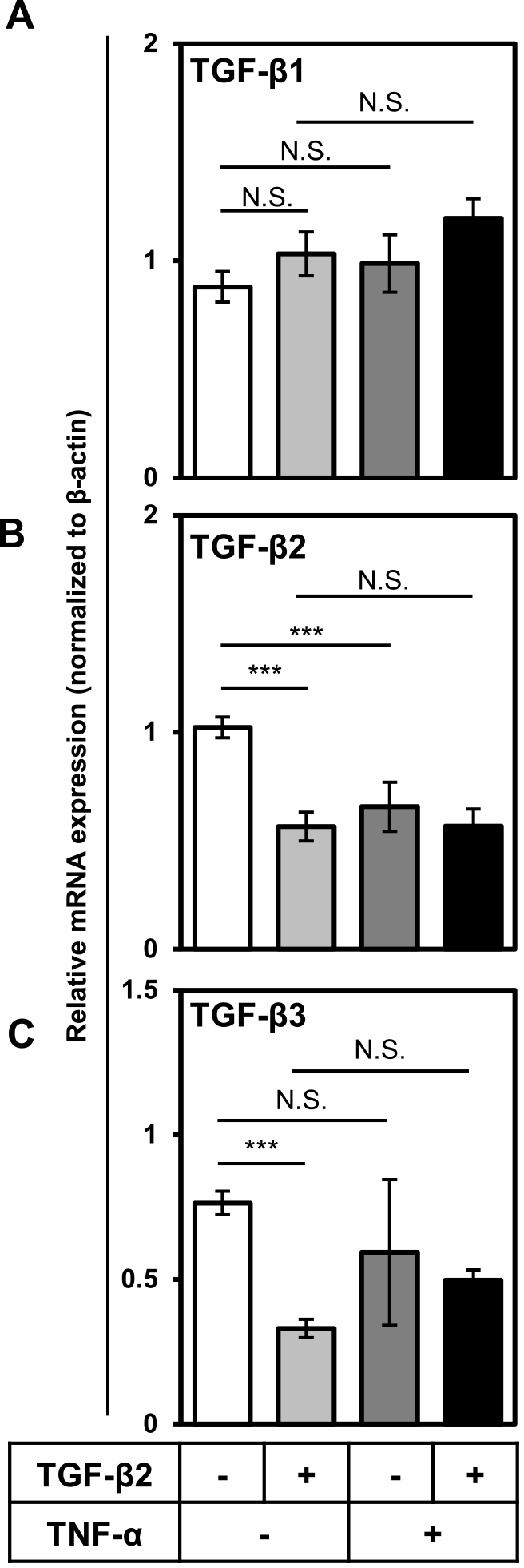

Supplement: S4 Fig — HDLECs were cultured in the absence (-) or presence (+) of 0.1 ng/mL of TGF-β 2 in combination with 10 ng/mL of TNF-α for 72 h, followed by qRT-PCR analyses for the expression of TGF-β 1 (A), TGF-β 2 (B), and TGF-β 3 (C). Data are represented as mean± S.D., N = 4, representative of three independent experiments. ***P < 0.001; N.S., not significant. Differences are tested using two-way ANOVA followed by Bonferroni multiple comparison post hoc analysis. (TIF) [file pone.0232356.s005.tif]

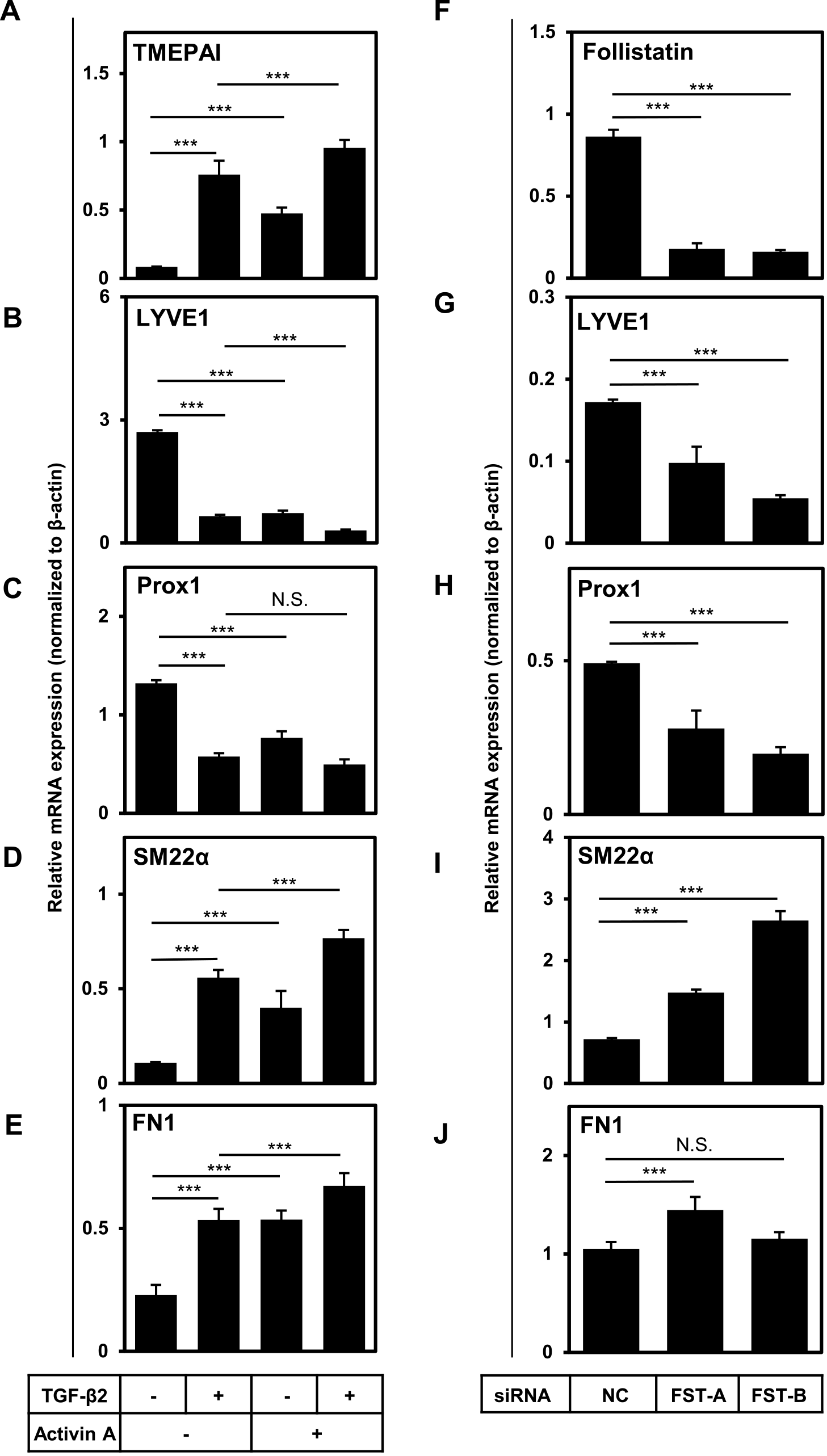

Supplement: S5 Fig — (A-E) HDLECs were cultured in the absence (-) or presence (+) of 0.1 ng/mL of TGF-β 2 in combination with 10 ng/mL of Activin A for 72 h, followed by qRT-PCR analyses for the expression of TMEPAI (A), LYVE1 (B), Prox1 (C), SM22α (D), and FN1 (E). (F-J) HDLECs transfected with negative control siRNA (NC) or siRNAs for Follistatin (FST-A and FST-B) were cultured for 48 h, followed by qRT-PCR analyses for the expression of Follistatin (F), LYVE1 (G), Prox1 (H), SM22α (I), and FN1 (J). Data are represented as mean± S.D., N = 4, representative of three independent experiments. *P < 0.05, **P < 0.01, ***P < 0.001; N.S., not significant. Differences are tested using two-way ANOVA (A-E) or one-way ANOVA (F-J) followed by Bonferroni multiple comparison post hoc analysis. (TIF) [file pone.0232356.s006.tif]

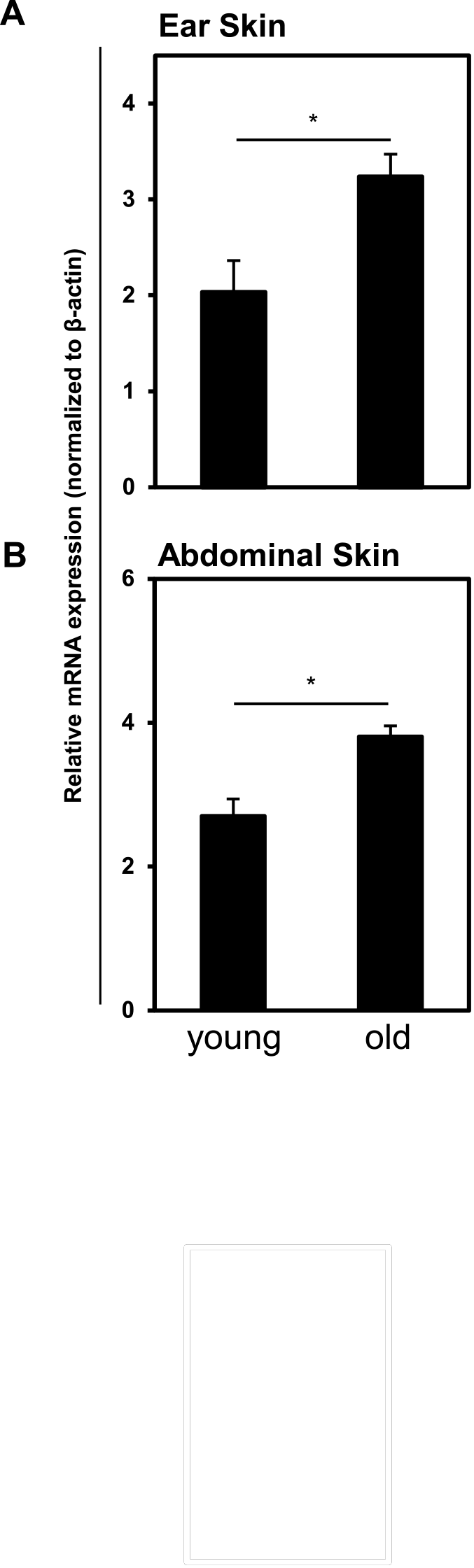

Supplement: S6 Fig — Ear and abdominal skin tissues were dissected from young (2 months) and aged (14–17 months) mice followed by qRT-PCR analyses for the expression of TGF-β 2 in ear skin (A) and abdominal skin (B). Data are represented as mean±S.E.M., N = 3, representative of two independent experiments. *P < 0.05. Differences are tested using Student t-test. (TIF) [file pone.0232356.s007.tif]
